# ATG4D is the main ATG8 delipidating enzyme in mammalian cells and protects against cerebellar neurodegeneration

**DOI:** 10.1038/s41418-021-00776-1

**Published:** 2021-04-01

**Authors:** Isaac Tamargo-Gómez, Gemma G. Martínez-García, María F. Suárez, Verónica Rey, Antonio Fueyo, Helena Codina-Martínez, Gabriel Bretones, Xurde M. Caravia, Etienne Morel, Nicolas Dupont, Roberto Cabo, Cristina Tomás-Zapico, Sylvie Souquere, Gerard Pierron, Patrice Codogno, Carlos López-Otín, Álvaro F. Fernández, Guillermo Mariño

**Affiliations:** 1 Departamento de Biología Funcional, Facultad de Medicina, Universidad de Oviedo, Oviedo, Spain; 2 Instituto Universitario de Oncología (IUOPA), Asturias, Spain; 3 Instituto de Investigación Sanitaria del Principado de Asturias (ISPA), Asturias, Spain; 4 Departamento de Bioquímica y Biología Molecular, Universidad de Oviedo, Oviedo, Spain; 5 Centro de Investigación Biomédica en Red de Cáncer (CIBERONC), Madrid, Spain; 6 U1151 and Institut Necker Enfants-Malades, INSERM, Sorbonne Paris Cité, Université Paris Descartes, Paris, France; 7 Departamento de Morfología y Biología Celular, Facultad de Medicina, Universidad de Oviedo, Oviedo, Spain; 8 Gustave Roussy Comprehensive Cancer Center, Villejuif, France; 9 CNRS, Villejuif, France

**Keywords:** Proteases, Macroautophagy, Disease genetics, Neural ageing, Neurological disorders

## Abstract

Despite the great advances in autophagy research in the last years, the specific functions of the four mammalian Atg4 proteases (ATG4A-D) remain unclear. In yeast, Atg4 mediates both Atg8 proteolytic activation, and its delipidation. However, it is not clear how these two roles are distributed along the members of the ATG4 family of proteases. We show that these two functions are preferentially carried out by distinct ATG4 proteases, being ATG4D the main delipidating enzyme. In mammalian cells, ATG4D loss results in accumulation of membrane-bound forms of mATG8s, increased cellular autophagosome number and reduced autophagosome average size. In mice, ATG4D loss leads to cerebellar neurodegeneration and impaired motor coordination caused by alterations in trafficking/clustering of GABA_A_ receptors. We also show that human gene variants of ATG4D associated with neurodegeneration are not able to fully restore ATG4D deficiency, highlighting the neuroprotective role of ATG4D in mammals.

## Introduction

Autophagy is a catabolic process, which has remained well conserved through evolution in all eukaryotic cells. In this process, portions of cytoplasm are sequestered by double-membrane vesicles called “autophagosomes”, which eventually fuse with lysosomes, thus acquiring hydrolytic enzymes responsible for degradation and recycling of macromolecules [1]. Multiple autophagy-related (ATG) proteins are required for the correct development of the process [2]. In yeast, one of the essential components for the elongation and expansion of nascent autophagosomes is the Atg4/Atg8 ubiquitin-like conjugation system, in which cytoplasmic protein Atg8 must be cleaved after a Gly residue by the cysteine proteinase Atg4 [3]. After this priming, Atg8 is conjugated with membrane-bound phosphatidylethanolamine (PE), allowing the correct formation, closure, and maturation of autophagosomes. Once Atg8 is not required at the autophagosomal membranes, Atg4 is also responsible for its delipidation, allowing its incorporation into newly formed autophagic structures. Contrasting to other autophagy-essential genes, the Atg4/Atg8 ubiquitin-like conjugation system has gained complexity throughout evolution. In fact, there are four human orthologues of Atg4 protease in mammalian cells (ATG4A-D) [4] and six Atg8 orthologues (mATG8s) have been described in humans, organized in two different subfamilies named as LC3 and GABARAP [5]. Although the specific functions of each ATG4 family member are not fully understood, it seems that ATG4B protease is the main priming enzyme for mATG8s [6]. ATG4C and ATG4A functions seem to be complementary and redundant to that of ATG4B [7], whereas ATG4D has been shown to prime and delipidate GABARAPL1 upon caspase-based activation in in vitro experiments [8]. Although most of the functions of ATG4 and mATG8 proteins are autophagy-related, some non-autophagic functions of mATG8 proteins are emerging. For example, LC3 proteins have been shown to regulate a GEF activity, whereas non-lipidated LC3B is able to regulate viral replication [9]. GABARAP proteins, especially GABARAP, also participate in other different cellular processes. Probably the most relevant of them is GABARAP participation in GABA_A_ receptor trafficking and clustering. GABA_A_ receptors constitute the main ligand-gated ion channels that respond to the inhibitory neurotransmitter gamma-aminobutyric acid (GABA). GABA receptors are normally a pentamer comprising two α‘s, two β‘s, and an additional variable subunit (either γ, δ, ε, π or π). There are six different types of α subunits (α_1-6_); three βs (β_1-3_) and three γs (γ_1-3_). The subunit composition of a determined GABA_A_ receptor will determine the receptor’s agonist affinity, chance of opening, conductance, and other properties [10, 11]. GABA_A_ receptor function and coordination and balance between inhibitory (GABAergic) and excitatory (glutamatergic) processes is essential for the correct development of neural functions [12]. In this work, through the analysis of ATG4D-deficient cells and *Atg4d*^−*/*−^ mice, we show that loss of ATG4D protease in mammalian cells leads to the accumulation of lipidated mATG8 proteins, which can be detected at the cytosolic leaflet of the external autolysosomal membranes. Moreover, we show that the loss of this protease does not impair autophagy flux, but alters autophagosomal dynamics, leading to increased cellular levels of autophagic structures, which are smaller in size than those found in *WT* cells. We also characterize the effects of *Atg4d* deletion in laboratory mice, which show motor coordination defects associated with a type of progressive cerebellar neurodegeneration different from that reported for other autophagy-deficient mouse strains. Remarkably, administration of the GABAA receptor agonist 4,5,6,7-tetrahydroisothiazolo-[5,4-c]pyridin-3-ol (THIP) is able to ameliorate the motor coordination defects of *Atg4d*^−*/*−^ mice in a variety of functional tests.

## Materials and methods

### Mice strains

Transgenic mice overexpressing GFP-LC3B were obtained from Dr. Noboru Mizushima (Tokyo Medical and Dental University, Tokyo, Japan). Atg4d-knockout mice were generated at University of Oviedo Facilities using the targeted ES cell clones (OST254045) that were obtained from Lexicon Genetics. Experimental groups consisted of littermates of different genotypes. When a treatment was used, mice were randomly assigned to each experimental group. Both females and males were used to rule out sex-dependent differences, and young (2-month-old) and old (15-month-old) mice were analyzed. All animal experiments were approved by the Committee on Animal Experimentation of Universidad de Oviedo (Oviedo, Spain), (PROAE 01/2017).

### Cell lines

The different MEF lines that were used in this work were generated as follows: MEFs were extracted from E13 embryos. Embryos were sterilized with ethanol, washed with PBS, and triturated with razor blades. Samples were then incubated in DMEM (Gibco) overnight at 37 °C and 5% CO_2_. The next day, cultured cells were trypsinized, filtered and washed. Finally, MEFs were incubated at 37 °C and 5% CO_2_ and used for the corresponding experiments. For CRISPR/Cas9-mediated gene targeting, MEFs and HEK293T cells were infected with the lentiviral vector LentiCRISPRv2 (Addgene repository). LentiCRISPRv2 vector was digested with BsmBI enzyme (Thermo Fisher). LentiCRISPRv2-ATG4D was generated by inserting 5′-TGGGGGTGCATGTTACGCAG-3′ and 5′-CTGCGTAACATGCACCCCCA-3′or 5′-TACGCAGCGGCCAGATGATG-3′ and 5′-CATCATCTGGCCGCTGCGTA-3′ hybridized oligonucleotides, respectively, in between BsmBI restriction sites of the LentiCRISPRv2 vector. LentiCRISPRv2-ATG4A was generated by inserting 5′-TGGGGATGTATGCTGCGcTG-3′ and 5′-CAGCGCAGCATACATCCCCA-3′ hybridized oligonucleotides, respectively, in between BsmBI restriction sites of the LentiCRISPRv2 vector. These cell lines were tested negative for mycoplasma contamination by PCR analysis.

### Antibodies

In this study, the following antibodies were used:

Anti-SQSTM1 (M01, clone 2C11) (Abnova Corporation, Cat# H00008878-M01); anti-LC3A (Proteintech, Cat# 12135-1-AP); anti-LC3B (Novus, Cat# NB600-1384); anti-LC3C (D3O6P) (Cell Signaling Technology, Cat# 14736); anti-GABARAP (MBL International, Cat# PM037); anti-GABARAPL1 (ATG8L) (Proteintech, Cat# 11010-1-AP); anti-GABARAPL2 (GATE-16) (MBL International, Cat# PM038); anti-GFP (Abcam, Cat# ab290); anti-beta-Actin (Sigma-Aldrich, Cat# A2228); anti-alpha-Tubulin (Sigma-Aldrich, Cat# T5168); anti-GAPDH (Novus Cat# NB300-320); anti-ubiquitin (FK2) (Enzo Life Sciences, Cat# BML-PW8810-0500); anti-GABA (A) receptor alpha1 (extracellular) (Alomone Labs, Cat# AGA-001); anti-GABA (A) receptor gamma2 (Novus Cat# NB300-151); anti-GABA(A) receptor delta (extracellular) (Alomone Labs, Cat# AGA-014); anti-Gephyrin (Synaptic Systems, Cat# 147 011C3); anti-Calbindin D28K (D-4) (Santa Cruz, Cat# sc-365360); anti-GFAP (Millipore, Cat# 04-1062); anti-NeuN (A60) (Millipore, Cat# MAB377); anti-LAMP-1 (H4A3) (Santa Cruz Biotechnology, Cat# sc-20011); anti-FIP200 (D10D11) (Cell Signaling Technology, Cat# 12436); anti-ULK1 (D8H5) (Cell Signaling Technology, Cat# 8054); anti-Phospho-ULK1 (Ser555) (D1H4) (Cell Signaling Technology, Cat# 5869); anti-phospho-ULK-1 (Ser757) (D7O6U) (Cell Signaling Technology, Cat# 14202); anti-Atg101 (E1Z4W) (Cell Signaling Technology, Cat# 13492); anti-Atg13 (D4P1K) (Cell Signaling Technology, Cat# 13273); anti-ATG14/Barkor (C-Terminal) (Proteintech, Cat# 24412-1-AP); anti-Beclin-1 (Cell Signaling Technology, Cat# 3738); anti-Atg12 (Cell Signaling Technology, Cat# 2011); anti-p70 S6 Kinase (Cell Signaling Technology, Cat# 9202); anti-Phospho-p70 S6 Kinase (Thr421/Ser424) (Cell Signaling Technology, Cat# 9204); anti-4E-BP1 (Cell Signaling Technology, Cat# 9452); Anti-AMPK-alpha (Cell Signaling Technology, Cat# 2532); anti-Phospho-AMPK (Thr172) (Cell Signaling Technology, Cat# 2531).

### Oligonucleotides

In this study, the following oligonucleotides were used:

LentiCRISPRv2-ATG4D targeting sequence:

5′-TGGGGGTGCATGTTACGCAG-3′; 5′-CTGCGTAACATGCACCCCCA-3′

5′-TACGCAGCGGCCAGATGATG-3′; 5′-CATCATCTGGCCGCTGCGTA-3′

LentiCRISPRv2-ATG4A targeting sequence:

5′-TGGGGATGTATGCTGCGCTG-3′; 5′-CAGCGCAGCATACATCCCCA-3′

Atg4a mouse genotyping (fragment size analysis)

5′-TTACCTTCACTCATTCCATTCCT-3′; 5′-TGGCTTGTCAGCCCATTACT-3′

Atg4b mouse genotyping:

5′-CCTCCAGCTCACTGAACTCC-3′; 5′-CACGCCATACAGTCCTCTTC-3′; 5′-AAGATATAGGCCTGGATGG-3′

Atg4c mouse genotyping:

5′-CCAACACATATTAGATGGAACCA-3′; 5′-TGACCGCTTCCTCGTGCTTTA-3′; 5′-CGGACTCTCGTGTCTTTACCTT-3′

Atg4d mouse genotyping:

5′-CAGACCGCAGGAAAGCAAGGTAT-3′; 5′-AAATGGCGTTACTTAAGCTAGCTTG-3′; 5′-AGTATAGAGTAACACTGTGCTGGC-3′

Atg4d human genotyping:

5′-GTGACATACAGCGTTTCCAG-3′; 5′-GCACCCCTTCCCCCCTATGG-3

### Plasmids and recombinant vectors

In this study, the following oligonucleotides were used:

LentiCRISPRv2 puro (Addgene_98290); psPAX2 (Addgene_12260); pMD2.G (Addgene_12259); pCI-neo-myc-LC3(deltaC22) (Addgene_45448); (Addgene_38193); pMXs-IP-EGFP-mAtg5 (Addgene_38196); pMRXIP GFP-Stx17 WT (Addgene_45909); pMRXIP GFP-VAMP8 (Addgene_45919). Other plasmids appearing in the paper which are not listed here were generated at our lab and are fully available upon request to the corresponding author.

### Other reagents

Other reagents and chemicals used in this study were:

Bafilomycin A1 (Enzo Life Science, Cat# BML-CM110-0100, CAS: 88899-55-2); Torin 1 (Selleckchem, Cat# S2827, CAS:1222998-36-8); 3-Methyladenine (Sigma-Aldrich, Cat# M9281, CAS: 5142-23-4); Baclofen (Sigma-Aldrich, Cat# B5399, CAS:1134-47-0); Gaboxadol hydrochloride (THIP) (Sigma-Aldrich, Cat# T101, CAS: 85118-33-8); Muscimol (Sigma-Aldrich, Cat# M1523, CAS: 2763-96-4); 1(S),9(R)-( − )-Bicuculline methiodide (Sigma-Aldrich, Cat# 14343, CAS: 40709-69-1).

### Atg4D-knockout mice generation

The targeted ES cell clones (OST254045) were obtained from Lexicon Genetics. They were grown, expanded, and subsequently injected into C57BL/6 blastocysts to generate chimeras. Chimeric males were mated with C57BL/6 female mice and the offspring heterozygous for *Atg4d* were used to generate homozygous null mice. In all experiments, homozygous *Atg4d*^−/−^ mice and their corresponding wild type (*WT*) controls were littermates derived from the interbreeding of heterozygotes with a mixed background of C57Bl6/129 Sv. In all cases, genotypes were determined by PCR analysis of tail DNA using a 5′ forward primer homologous to DNA sequence upstream exon 1 (5′-AGTATAGAGTAACACTGTGCTGGC-3′), a 3′ reverse primer from exon 1 (5′-CAGACCGCAGGAAAGCAAGGTAT-3′) to observe the *WT* allele, and a 3′ reverse primer (5′-AAATGGCGTTACTTAAGCTAGCTTG-3′) from the LTR region of the PGK-Neo cassette. A 500 bp fragment was generated in the presence of the *WT* allele and a 220 bp fragment in the presence of the targeted allele.

### Analysis of GFP-LC3B mice tissues

Transgenic mice overexpressing GFP-LC3B were perfused with 4% paraformaldehyde in 0.1 M PBS, pH 7.4. After that, tissues were harvested and fixed with the same fixative solution for at least 4 h, followed by treatment with 15% sucrose in PBS for 4 h, and then with 30% sucrose solution overnight. Tissue samples were embedded in Tissue-Tek OCT compound (Sakura Finetechnical Co. Ltd.) and stored at –70 °C. Samples were then sectioned at 5 µm thickness with a cryostat (CM3050 S, Leica), air-dried for 1 h, washed in PBS for 5 min, dried at room temperature for 30 min, and mounted with a conventional anti-fading medium. GFP-LC3B dots were observed in five independent visual fields from at least five independent tissues per genotype/condition.

### Histology analyses

Histological analyses were performed on formalin-perfused, paraffin-embedded sections. Hematoxylin–eosin (HE) was performed to examine the morphology of the cerebellum. GFAP alteration was evaluated according to the following score: - Grade 0: Without alterations; - Grade 1: Focal gliosis; - Grade 2: Diffuse gliosis All tissues were examined by a pathologist in a blinded fashion.

### Stereological quantification of the number of neuronal nucleus profiles per unit of area

Quantification of Purkinje cell number was performed by using the following method, as previously described [13]:

NA = (∑Q)/(n × a/f) = (∑Q)/(n × 0,001) = mm-2

∑Q: Number of positive Purkinje (NeuN) nuclei profiles that fall within the counting frames.

n: Number of counting frames used to count the nuclear profiles of positive Purkinje neurons (NeuN)

a/f (area associated with each frame): (∆X x ∆X)/M² = (441)/302500 = 0,001 mm2

M (magnification): (11 mm)/20microns = M (magnification): (11 mm)/(0,02 mm) = 550

∆x: 21 mm

### Immunofluorescence analyses

For immunofluorescence analyses with tissue cryo-sections, sections were pretreated for 30 min in 1% H_2_O_2_/PBS, followed by 1 h in blocking solution and incubated overnight with mouse anti-calbindin D28K (sc-365360; Santa Cruz Biotechnologies). Peroxidase activity was developed with the Elite Vectastain kit (Vector Laboratories) using diaminobenzidine (Dako). Sections were coverslipped with PermaFluor Aqueous Mounting Medium (Thermo Scientific). Digital images were captured with a Nikon eclipse 80i optical microscope using the software NIS-Elements Basic Research. For paraffin sections, slides were deparaffinized and rehydrated. Slides or wells were blocked in 10% goat serum for 10 min, incubated with primary antibodies overnight at 4 °C, washed in PBS, incubated for 40 min with secondary antibodies, thoroughly washed in PBS, and stained with DAPI for nuclear staining. For immunofluorescence analyses of MEFs, cells were grown 96-well black clear tissue culture-treated plates, washed in PBS, and fixed in 4% paraformaldehyde in PBS at room temperature for 10 min. Primary antibody was diluted 1:100 in PBS, incubated overnight at 4 °C. Samples were washed three times in PBS for 15 min each. Secondary antibody was diluted 1:300 in PBS, incubated at RT for 1 h. Samples were washed three times in PBS for 15 min each and analyzed by fluorescence microscopy.

### In vivo analysis of motor functions

All in vivo analyses were performed with male or female mice aged 2–15 months. For all analysis, evaluators were unaware of either the genotype or the injected drug until analyses were completed. Except for Bicuculline, the effect of all drugs used in this work was evaluated 30–45 min after intraperitoneal administration. For bicuculline, mice were injected daily during 1 week and the experiment was done one day after the last dose. At least six mice were used for each experimental group in this type of analyses. THIP (Sigma-Aldrich) was used in a working concentration of 1.25 mg/kg for rotarod and raised beam test and 2.5 mg/kg for tail suspension test. Bicuculline (Sigma-Aldrich) was used in a working concentration of 0.75 mg/kg for rotarod, raised beam test and for tail suspension test. Baclofen (Sigma-Aldrich) was used in a working concentration of 3 mg/kg for rotarod, raised beam test and for tail suspension test. Muscimol (Sigma-Aldrich) was used in a working concentration of 0.75 mg/kg for rotarod, raised beam test and tail suspension test.

### Footprint analyses

Hindlimbs were dipped into red non-toxic paint, whereas whereas forelimbs were painted blue. Mice were allowed to walk through a plexiglass tunnel where the floor of which was covered with a sheet of white paper (20 × 50 cm). Stride length for each mouse was analyzed.

### Tail suspension test

Mice were suspended by their tails and evaluated after limb positions were stabilized (normally within 60 s). Positions were photographically captured laterally to measure the angle of the upper forelimb against the body axis.

### Raised-beam test

Mice were acclimated to crossing a 100 cm-long wooden square beam (8 mm width) elevated 30 cm above a padded base. Mice were placed on the start platform and allowed to traverse the beam to the opposite end. The time needed to cross the entire length of the beam was measured and the number of paw slips was counted. One (1) point was awarded to each paw slip. Two (2) points were awarded to each mouse fall while the test was developed. Animals were videotaped while traversing the wooden square beam for a total of three trials. A blinded observer viewed videotapes and analyzed the data.

### Rotarod test

An accelerating rotarod LE8500 (LSI, LETICA) was used to evaluate the neuronal response of *Atg4d*^−/−^ mice. The rod accelerated from 4 to 40 rpm in 5 min and remained at maximum speed for the next 5 min. Animals were scored for their latency to fall (in seconds) for each of the six trials and rested a minimum of 10 min between trials to avoid exhaustion and fatigue.

### Grip strength test

For the grip strength test, mice were placed on a cage lid with a wire grid which is then inverted over a padded surface. The total time the animal supports its weight before falling was recorded, and the final score was calculated as the average time of three different trials (with 15 min resting periods in between).

### Open field and novel object recognition test

Tests were performed as previously described [14]. Briefly, mice were placed in the center of a rectangular Plexiglass arena (60 × 38 × 19.5 cm) and let to freely explore for 5 min. This habituation phase was used as an open field test.

Twenty-four hours after the habituation phase, mice were placed in the same arena, where two identical objects were located in opposite quadrants (objects A and A1), and allowed free exploration for 5 min, as a training phase. Next, long-term memory (LTM) was evaluated 24 h later, to explore LTM, and object A1 was changed by a new object, B. Mouse behavior was recorded by a zenithal webcam (HD Webcam C270, Logitech, Lausana, Switzerland) connected to a computer. The arena was cleaned with 70% alcohol after each mouse. Three researchers, blind to both genotype and group, visualized the videos and the number of times a mouse interact with an object was noted. Thus, LTM recognition index was calculated by the formula [(B-A)/(A + B)] *100.

### Lentiviral transduction of MEF cells

HEK293T- cells were maintained in Dulbecco’s modified Eagle’s medium (DMEM, Sigma-Aldrich) supplemented with 10% fetal bovine serum, 1% penicillin-streptomycin-l-glutamine and 1% antibiotic- antimycotic (Gibco) at 37 °C in 5% CO2. In the case of mouse fibroblasts, ×1 non-essential amino-acids, 10 mM HEPES buffer, 100 μM 2-mercaptoethanol and ×1 sodium pyruvate (Gibco) were also added to the culture medium and 10% fetal bovine serum was used. Transfections were carried out in cells seeded onto gelatinized coverslips and using lipofectamine plus or Lipofectamine 3000 (Invitrogen), following the manufacturer’s instructions. For lentiviral infection, HEK-293T cells were transfected with lentiviral vector together with packaging plasmids psPAX2 and pMD2.G using Lipofectamine 3000 (Invitrogen). 48 h post-transfection, supernatants were filtered through 0.45 μm polyethersulfone filters to collect the viral particles and added at 1:3 dilution to previously seeded mouse fibroblasts supplemented with 0.8 µg/ml of polybrene (Millipore). Selection with puromycin (2 µg/mL) (Sigma-Aldrich) was performed 2 days after infection. For immortalization, pLOX-Ttag-iresTK (Addgene_ 12246) plasmid was used. Briefly, primary MEFs between passage 3 and 4 were split into 6 well dishes with 2 ml MEF culture medium and grown overnight in a 37 °C incubator with 5% CO2. Once the MEFs reached ~70% confluence, cells were considered ready for infection. The viral supernatant was removed after 48 h and replaced with 2 ml MEF culture medium then incubated at 37 °C until just confluent, ~48–72 h after infection. Once confluent, the MEFs were sub-cultured into a 10 cm tissue culture dish with 10 ml MEF culture medium this was considered passage one (P1). The MEFs were split every 3–4 days for 10–15 days after P1 until its immortalization. An uninfected Primary MEFs (negative control) is used to know which passage implies immortalization.

### RT-PCR

Total RNA was isolated from mouse tissues according to the method of Chomczynski and Sacchi [15]. About half of the obtained product was reverse-transcribed using the RNA-PCR Core kit^®^ from Perkin-Elmer (Roche Applied Science, Indianapolis, IN). A PCR reaction was then performed with mouse *Atg4d* specific primers for 25 cycles of denaturation (94 °C, 20 s), annealing (62 °C, 20 s), and extension (72 °C, 30 s). As a control, actin was PCR-amplified from all samples under the same conditions.

### Quantitative real-time PCR

cDNA was synthesized using 1–5 µg of total RNA, 0.14 mM oligo(dT) (22-mer) primer, 0.2 mM concentrations each of deoxynucleoside triphosphate and SuperScript II reverse transcriptase (Invitrogen). Quantitative RT-PCR (qRT-PCR) was carried out in triplicate for each sample using 20 ng cDNA, TaqMan Universal PCR Master Mix (Applied BioSystems) and 1 μl of the specific TaqMan custom gene expression assay for the gene of interest (Applied Biosystems). To quantify gene expression, PCR was performed at 95 °C for 10 min, followed by 40 cycles at 95 °C for 15 s, 60 °C for 30 s and 72 °C for 30 s using an ABI Prism 7700 Sequence Detection System. As an internal control for the amount of template cDNA used, gene expression was normalized to the mouse β-actin gene using the Mouse β-actin Endogenous Control (VIC/MGB Probe, Primer Limited). Relative expression was calculated as RQ = 2^−ΔΔCt^.

### Protein extract preparation

Tissues were immediately frozen in liquid nitrogen after extraction and homogenized in a 20 mM Tris buffer pH 7.4, containing 150 mM NaCl, 1% Triton X-100, 10 mM EDTA and Complete^®^ protease inhibitor cocktail (Roche Applied Science). Then, tissue extracts were centrifuged at 12.000 rpm at 4 °C and supernatants were collected. Protein concentration was quantified by bicinchoninic acid technique (BCA protein assay kit, Pierce Biotechnology, 23225). For protein extracts derived from cultured cells, cells were washed with cold PBS and lysed in a buffer containing 1% NP-40, 20 mM HEPES (pH 7.9), 10 mM KCl, 1 mM EDTA, 10% glycerol, 1 mM orthovanadate, 1 mM phenylmethanesulfonyl fluoride, 1 mM dithiothreitol, 10 µg/ml aprotinin, 10 µg/ml leupeptin and 10 µg/ml pepstatin. Lysates were centrifuged at 12.000 *rpm* at 4 °C and supernatants were collected. Protein concentration was quantified by bicinchoninic acid technique (BCA protein assay kit, Pierce Biotechnology, 23225).

### Immunoblotting

A total of 25 µg of protein sample was loaded on either 8% or 13% SDS-polyacrylamide gels. After electrophoresis, gels were electrotransferred onto polyvinylidene difluoride membranes (Millipore), and then membranes were blocked with 5% non-fat dried milk in PBT (phosphate-buffered saline with 0.05% Tween 20) and incubated overnight at 4 °C with primary antibodies diluted in 3% non-fat dried milk in PBT. After three washes with PBT, membranes were incubated with the corresponding secondary antibody at 1:10.000 dilution in 1.5% milk in PBT and were developed with Immobilon Western Chemiluminescent HRP substrate (Millipore, P36599A) by using Odyssey^®^ Fc Imaging System (LI-COR, Lincoln, NE, USA). Unless otherwise specified, immunoblotting against β−actin was used as sample processing control (LOAD) for the immunoblots shown in this article.

### Co-immunoprecipitation

For immunoprecipitation assays in mouse cerebellum, protein samples were obtained using a lysis buffer containing 25 mM HEPES (pH 7.4), 25 mM KCl, 1 mM EDTA, 1 mM EGTA, 500 µM DTT, 0.5% Triton X-100 and Complete^®^ protease inhibitor cocktail (Roche Applied Science). Samples were pre-cleared with Protein A/G-PLUS agarose beads (1:25, Santa Cruz) for 1 h at 4 °C and then incubated overnight with anti-rabbit GABARAP antibody (MBL International, Cat# PM037, 1:500) and agarose beads. The following day, these beads were pelleted, washed, mixed with loading buffer, and boiled for 15 min. Finally, the supernatants were resolved on SDS-PAGE. The same protocol was used for immunoprecipitation assays in HEK293T cells overexpressing HA-GABARAP and GABA_A_Rγ2-GFP proteins, incubating the samples with GFP-Trap^®^ agarose beads (Chromotek, cat# gta) for 2 h to precipitate the receptor.

### Fluorescence microscopy

Fluorescence microscopy images were acquired ﻿with an Axio Observer Z1 platform with a Plan-Apochromat 40X/1.3, (working distance, 0,21 mm) equipped with an ApoTome.2 system and an Axiocam MRm camera (from Carl Zeiss, Jena, Germany). Zeiss Immersol^®^ immersion oil was used for all microscopic analyses. mKeima-LC3B ratio determination and colocalization of mKeima-LC3B (Ex586/Ex440) were measured with Spots colocalization (ComDet) ImageJ plugin.

### Specific labelling of SNAPtag®-LC3B at the cytosolic leaflet of the outer autophagosomal/autolysosomal membrane

SNAPtag^®^-LC3B expressing cells were grown in the indicated conditions and then treated with 1× MAS buffer (220 mM mannitol, 70 mM sucrose, 10 mM KH2PO4, 5 mM MgCl2, 2 mM HEPES, 1 mM EGTA) containing perfringolysin (XF-MPM^®^) to permeabilize the plasma membrane (PM) and release SNAPtag^®^-LC3B-I from the cytosol for 15 min at 37 °C. After, cells were washed three times with 1× MAS buffer. Then, cells were incubated with a fluorescent Membrane Impermeable SNAP-Surface^®^ Ligand (MIL) to stain membrane-bound SNAPtag^®^-LC3B-II facing the cytosol. Next, cells were then fixed and immunostained for LAMP-1 lysosomal protein. This allows the specific detection of autolysosomes positive for SNAPtag^®^-LC3B-II at their cytosolic membrane leaflet (MIL^+^/LAMP1^+^). After completing the procedure, LAMP1/MIL colocalization in cells was measured with Spots colocalization (ComDet) ImageJ plugin.

### GFP-LC3 degradation assay by flow cytometry

Primary MEFs at 80% confluency from stably expressing GFP-LC3B *WT* and Atg4d-deficient mice were incubated in Dulbecco’s modified Eagle’s medium (DMEM, Sigma-Aldrich) complemented with heat-inactivated fetal bovine serum as control condition; in amino acid-free Earle’s balanced salt solution (EBSS, Sigma-Aldrich) to induce autophagy and in EBSS with Bafilomycin A1 (50 nM, Enzo Life Science) to block the autophagy flux. After 4 h of incubation, cells were trypsinized, pelleted by centrifugation, washed with Dulbecco’s Phosphate-Buffered Saline (DPBS), and pelleted again. Cell pellets were resuspended in DPBS supplemented with 5% of heat-inactivated FBS to a density of 105 cells/mL, kept in ice and GFP fluorescence intensity immediately analyzed by Fluorescence-Activated Cell Sorter (BD FACSAria Il, BD Biosciences). Data analysis and graphical representation were done using the free software Flowing Software 2.5.1. The level of the GFP fluorescence intensity was normalized to the level of the control sample, set at 100%. Data represent the mean and SEM of three independent experiments.

### CYTO-ID^®^ assay

MEFs from *WT* and Atg4d-deficient mice were incubated in Dulbecco’s modified Eagle’s medium (DMEM, Sigma-Aldrich) as control and upon two different autophagy-inducing conditions – Torin (Selleckchem, at 250 nM) and EBSS (Sigma-Aldrich)- in a 96 well plate. After 4 h of treatment, CYTO-ID^®^ detection kit (Enzo Life Science, ENZ-51031) was used to stain the autophagic vesicles according to the product manual. Briefly: treatment media was removed; cells were washed with buffer and the detection solution then added. After 30 min of incubation at 37 °C, cells were washed twice with buffer and immediately analyzed in a BD Pathway 435 System (BD Bioscience).

### Automated fluorescence microscopy

MEFs cells stably expressing fluorescent markers were seeded in 96-well imaging plates (BD Falcon, Sparks, USA) 24 h before stimulation. Cells were treated with the indicated agents for 4 h. Subsequently, cells were fixed with 4% PFA and counterstained with 10 µM Hoechst 33342. Images were acquired using a BD pathway 435 automated microscope (BD Imaging Systems, San Jose, USA) equipped with a 40× objective (Olympus, Center Valley, USA). Images were analyzed for the presence of fluorescent puncta in the cytoplasm by means of the BD Attovision software (BD Imaging Systems). Cell surfaces were segmented and divided into cytoplasmic and nuclear regions according to standard proceedings. RB 2 × 2 and Marr-Hildreth algorithms were used to detect cytoplasmic GFP-LC3 positive dots.

### Transmission electron microscopy

For ultrastructural studies, cultured cells were fixed in 1.6% glutaraldehyde (v:v in 0.1 M phosphate buffer) for 1 h, scraped off the plastic dish, centrifuged and post-fixed as a cell pellet in 1% osmium tetroxide (w:v in 0.1 M phosphate buffer) for 2 h. Following dehydration through a graded ethanol series, cells were embedded in EponTM 812. Ultrathin sections were stained with standard uranyl acetate and lead citrate. For immunogold studies, cells were fixed with either 4% formaldehyde or 1.6% glutaraldehyde in 0.1 M phosphate buffer (pH 7.3) for 1 h at 4 °C. Cell pellets were dehydrated in methanol and embedded in Lowicryl K4M at −20 °C in an AFS2 Freeze Substitution Processor apparatus (Leica Microsystems). Polymerization under UV light was carried out for 2 days at −20 °C, followed by 2 days at 20 °C. Ultrathin sections were incubated with primary antibodies specific to GFP (#ab290, Abcam), for 1 h at room temperature, and then with secondary antibodies conjugated to 10–15 nm gold particles (BBI International, Cardiff, UK), as appropriate. Images were acquired with a Tecnai 12 electron microscope (FEI, Eindhoven, the Netherlands). For electron microscopy of mice tissues, tissue samples were harvested from mice and immediately fixed in 3% glutaraldehyde in 0.1 M sodium cacodylate (pH 7.2) overnight. After 3 washes in 5% sucrose in 0.1 M sodium cacodylate buffer, samples were post-fixed with 1% osmium tetroxide for 1 h in darkness and rinsed three times in 0.1 M sodium cacodylate. Tissues were dehydrated with increasing acetone concentrations: 30% for 10 min, 60% for 10 min, 90% for 10 min and 100%, three times, for 10 min each. The dehydrated pieces were then immersed in mixtures of anhydrous acetone and resin (Durcapan^(R)^ ACM, Fluka BioChemika) of increasing resin concentrations (1:1, 1:2) each step for 30 min, and then in pure resin (at 37 °C for 12 h following of 60 °C for 24 h). Ultrathin sections (85 nm) were taken from each sample and analyzed on a Jeol (JEM-1011).

### Proteolysis assay

Assessment of long-lived protein degradation by autophagy was performed as described previously. Briefly, cells were incubated at 37 °C with 0.2 μCi/ml of L-[14 C] valine for 18 h and free radioisotopes were removed by three rinses with PBS. Then, cells were cultured with fresh supplemented medium for 1 h to degrade short-lived proteins and, when required, 3-MA was added to inhibit new autophagosome formation. Next, autophagy was induced if needed by 4 h incubation in Earl’s Balanced Salt Solution (EBSS). The medium was then precipitated overnight with 10% trichloroacetic acid (TCA) and the acid-soluble radioactivity was measured by liquid scintillation counting after centrifugation (10 min at 470 g at 4 °C). In parallel, cells were washed twice with cold 10% TCA, the cell pellet was dissolved in 0.2 M NaOH for 2 h and radioactivity measured. Degradation rate was calculated from the ratio of medium acid-soluble radioactivity to that in acid-precipitable cell fraction.

### Computer-based 3D Structure modeling of ATG4D

The Protein Data Bank (PDB) contains one structure for ATG4A (2p82.pdb). It also contains several structures for ATG4B; we chose 2zzp.pdb [Satoo & Inagaki 2009 the structure of Atg4B-LC3 complex (2zzp) reveals the mechanism of LC3 processing and delipidation during autophagy.pdf] because it contains the protease complexed to one of its substrates (rat LC3B), and because its engineered mutation (catalytic Cys to Ser) was more conservative than the engineered mutations in other complexes (catalytic His to Ala). We downloaded both structures from PDB_REDO [Touw & Vriend 2016 structure validation and PDB_REDO.pdf], a database where the structures originally deposited in the PDB are re-refined and re-built with up-to-date software. We aligned structures and sequences with SALIGN, corrected the alignment manually, and predicted the structures with the PyMod interface for MODELLER 9.19. We calculated residue pKas with PROPKA. Free energy changes with FoldX 4. Electrostatic values with PDB2PQR using the AMBER force field, and APBS. We created the images with PyMOL [https://pymol.org/2/support.html?#citing].

### Quantification and statistical analysis

All data acquisition and analyses were performed by investigators blinded to experimental group. For biochemical analyses, a minimum of four samples per genotype were used for each analysis, while in vivo analyses included at least six mice per genotype. These sample sizes are sufficient to determine whether there is a biologically meaningful difference between different genotypes, given the known mouse-to-mouse variation in autophagy assessments in previous studies published over the past decade. As for in vitro studies, a sufficient large number of cells/areas were analyzed to ensure the description of biologically meaningful differences, also following the methods from studies cited throughout the paper. Moreover, results obtained in cells were reliably reproduced in at least three independent experiments. All experimental data are reported as mean ± SEM unless otherwise mentioned. Normality of the variables was tested by means of the Shapiro–Wilk test. The data from the analyses met the assumptions of the tests and the variance was similar between the experimental groups. Unpaired two-tailed Student´s *t* test was used when comparing two experimental groups, while three experimental groups were analyzed using *one-way ANOVA* followed by Tukey’s post hoc test. Analysis with *repeated-measures ANOVA* (used to address the performance of the same animal before and after drug administration) or *two-way ANOVA* (used when four experimental groups and two variables were analyzed) were followed by Dunnett’s post hoc test. Chi-square was applied for soma alignment analysis. The Prism program version 7.0 (Graph-Pad Software Inc.) was used for calculations and *P* values lower than 0.05 were considered significant.

## Results

### Generation and development of *Atg4d*^−*/*−^ mice

**Fig. 1:**
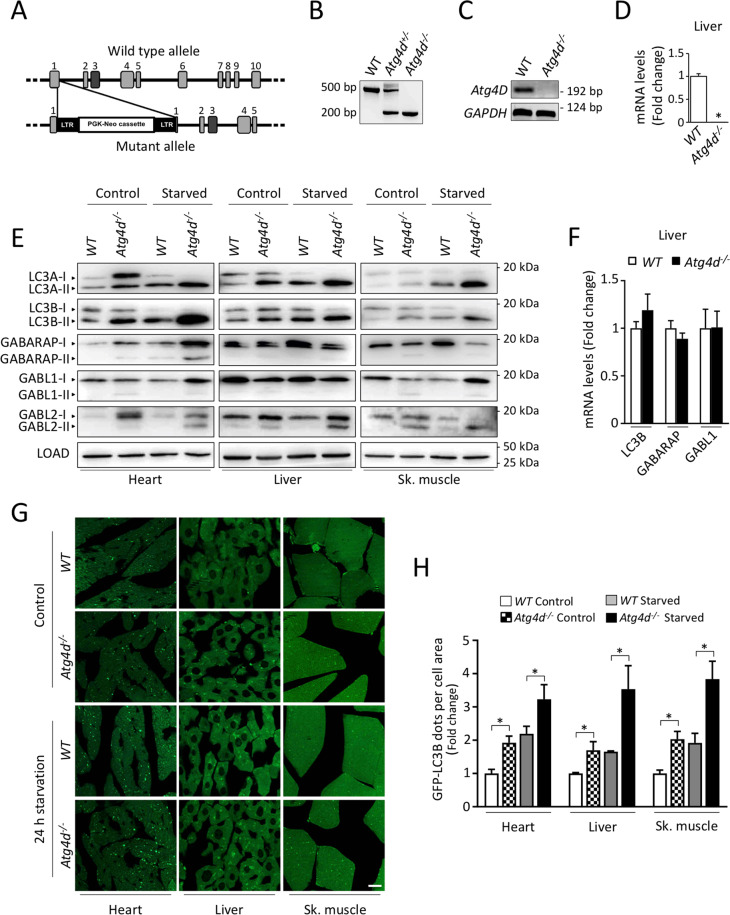
Generation and characterization of *Atg4d*-deficient mice. **A** Up, schematic representation of *WT Atg4d* locus, with coding exons represented as numbered boxes. Bottom, schematic representation of the mutant allele, showing the insertion of a PGK-Neo cassette in exon 1, disrupting the transcription of the gene. **B** PCR analysis of genomic DNA from *WT*, heterozygous and *Atg4d*-null mice. **C** RT-PCR analysis of RNA of muscle tissue from control and *Atg4d*^*−/−*^ animals showing the absence of full-length *Atg4d* mRNA expression in mutant mice. **D** qPCR analysis of liver tissue RNA from *WT* and *Atg4d* knockout mice confirming the absence of *Atg4d* expression. **E** Representative immunoblots of endogenous ATG8-like proteins in extracts from control and knockout mice tissues. Mice were fed ad libitum or fasted for 24 h, and GAPDH were used as sample processing controls. *N* = 6 mice per genotype and condition. **F** Quantitative RT-PCR analysis of LC3B, GABARAP and GABARAPL1 mRNA in liver tissue. **G** Representative images of tissue sections from age-matched *WT* and *Atg4d*^*−/−*^ mice stably expressing the GFP-LC3B transgene, either fed ad libitum or after 24 h of starvation. Scale bars, 10 µm. **H** Quantification of the data shown in (**G**). Bars represent means ± SEM (*N* > 3 mice per genotype and condition). Scale bars, 10 µm. *P < 0.05, 2-tailed unpaired Student’s *t* test.

To assess the in vivo functions of the ATG4D cysteine proteinase we generated mice with a targeted mutation in the gene encoding this enzyme (Fig. 1A). PCR analysis verified the homozygosity for the mutation (Fig. 1B), and the absence of *Atg4d* transcript was demonstrated by both RT-PCR and qPCR (Fig. 1C, D). Despite this deficiency, *Atg4d*-null mice had normal embryonic development and reached adulthood, with males and females being fertile. Plasma levels of major metabolites and the abundance of white and red blood cells was comparable between age-matched *WT* and mutant mice (Fig. S1A-B).

### *Atg4d*^*−/−*^ mice show accumulation of lipidated mammalian ATG8-like proteins

To evaluate if the absence of ATG4D has any impact in the dynamics of the autophagic process in mice, we first analyzed the status of the murine orthologues for yeast Atg8, mATG8s, which are ATG4D putative substrates (LC3A, LC3B, GABARAP, GABARAPL1 and GABARAPL2). As shown in Fig. 1E, immunoblotting analyses revealed an increased content of the membrane-bound forms of most mATG8s in *Atg4d*^*−/−*^ tissues, either fed ad libitum or upon 24 h of fasting which were not attributable to changes in their mRNA expression (Fig. 1E-F and S1D). To extend these analyses, we crossed these mutant mice with those expressing the transgene GFP-LC3B, that provides an efficient in vivo marker for autophagosomes [16]. Fluorescence microscopy analyses showed a significant increase of GFP-LC3B puncta, either fed ad libitum or upon 24 h of fasting in liver, heart, and skeletal muscle (Fig. 1G-H).

### Autophagy flux is not altered in the absence of ATG4D

**Fig. 2:**
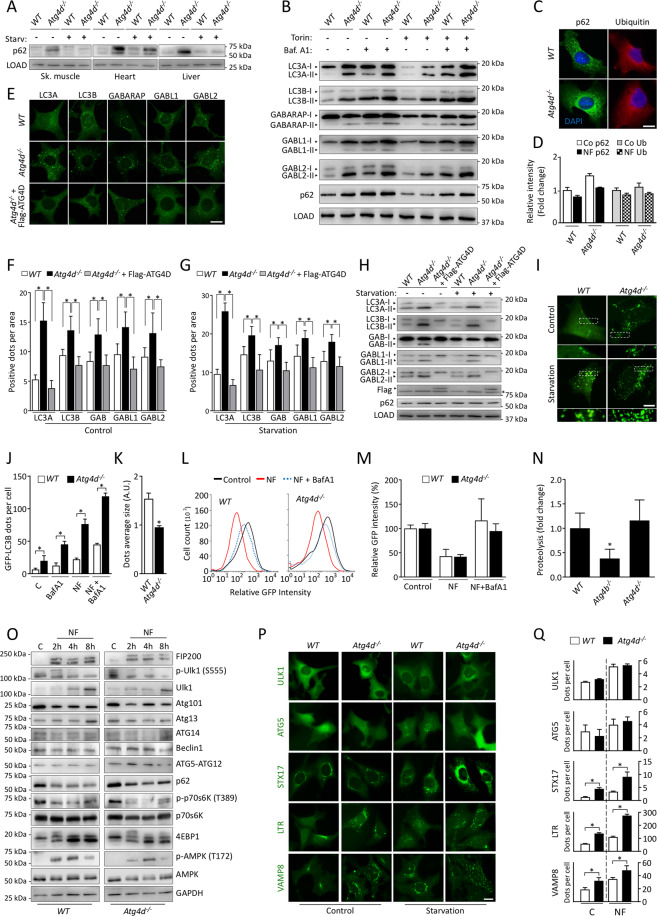
Autophagy regulation and flux analyses in *Atg4d*^*−/−*^ mice. **A** Representative immunoblotting of SQSTM1/p62 in protein extracts from *WT* and mutant mice showing the effect of 24 h starvation on SQSTM1/p62 degradation. α-tubulin (skeletal muscle and heart) or β-actin (liver) were used as sample processing controls. *N* = 6 mice per genotype and condition. **B** Immunoblotting analyses against mATG8 proteins showing that the higher content of their lipidated forms in *Atg4d*^*−/−*^ MEFs is not due to autophagic flux block. Torin1 was used as autophagy inducer and BafA1 was used to inhibit lysosomal degradation of mATG8-membrane-bound forms. **C** Representative immunofluorescence images of endogenous SQSTM1/p62 and Ubiquitin in *WT* and knockout MEFs. Cells cultured in full medium are shown in the images. **D** Quantification of the data shown in (**C**) showing the effect of nutrient deprivation on p62 degradation and Ubiquitin accumulation in cultured MEFs (Co: Full medium and NF: Nutrient-free medium). **E** Representative images of immunofluorescence analysis of endogenous mATG8 proteins in *WT*, *Atg4d*^−/−^ MEFs and *Atg4d*^−/−^ MEFs expressing Flag-ATG4D. Images show cells cultured in full medium. **F, G** Quantification of the data from (**E**) either in Control (**F**) or upon Nutrient deprivation (**G**). **H** Immunoblotting analyses of mATG8 proteins in *WT*, *Atg4d*^*−/−*^ MEFs and *Atg4d*^*−/−*^ MEFs stably expressing Flag-ATG4D in the indicated conditions. **I** Representative fluorescence microscope images of *WT* and mutant MEFs stably expressing GFP-LC3B in basal and autophagy-inducing conditions. **J–K** Quantification of average GFP-LC3B content (**J**) and average GFP-LC3B dot size (**K**) from the data shown in (**I**). **L** Representative flow cytometry profiles for GFP-LC3B degradation in response to nutrient deprivation in the presence/absence of BafA1. **M** Quantification of the data from (**L**). Average *WT* values were set to 100%. **N** Autophagic-dependent degradation (sensitive to the autophagy inhibitor 3-MA) of isotope-labelled long-lived proteins upon nutrient deprivation in a pulse-chase analysis. **O** Immunoblotting analyses of the major autophagy-regulatory pathways in *WT* and *Atg4d*^*−/−*^ MEFs upon nutrient deprivation. **P** Representative images of *WT* and *Atg4d*^*−/−*^ MEFs stably expressing GFP-ULK1, GFP-ATG5, GFP-STX17TM, GFP-VAMP8 or stained with Lysotracker^(R)^ were cultured in regular or starvation medium for 4 h (NF). **Q** Quantification of the data represented in the images. LOAD: GAPDH (skeletal muscle, heart and liver) or β-actin (cells). Bars represent mean ± SEM (*N* > 80 cells per condition). Scale bars, 10 µm. *P < 0.05, 2-tailed unpaired Student’s *t* test (in **D**, **J**, **K**, **M** and **Q**) and one-way ANOVA followed by Tukey´s post hoc test (in **F**, **G** and **N**).

To further characterize the effects of ATG4D deficiency in autophagic degradation, we analyzed the specific autophagic substrate p62/SQSTM1. As shown in Fig. 2A and S2A, the levels of this protein were increased in tissue samples from mutant mice, which was not attributable to changes in mRNA synthesis (Fig. S1C). Interestingly, p62/SQSTM1 levels decreased in knockout mice tissues upon nutrient deprivation, suggesting that starvation-induced autophagy is not compromised in the context of ATG4D deficiency (Fig. 2A and S2A).

Consistently, autophagic flux analysis in MEFs confirmed that the increased lipidation of mATG8s is not a consequence of autophagic flux blockade, as the increase in mATG8s-II levels upon BafA1 treatment both in control conditions or upon Torin treatment is comparable in WT and *Atg4d*^−/−^ cells, as well as starvation-induced p62/Ubiquitin degradation (Fig. 2B-D and S2B). Immunofluorescence analyses revealed that ATG4D-deficient MEFs present an increase in the number of dot-like structures positive for all mATG8s (Fig. 2E-G). As expected, re-expression of ATG4D in *Atg4d*^−/−^ cells restored these defects (Fig. 2E-H and S2C). Moreover, CRISPR/Cas9-based targeting of human ATG4D in HEK293 cells resulted in a similar phenotype to that observed in *Atg4d*^−/−^ MEFs (Fig. S3).

MEFs derived from *Atg4d*^*−/−*^*/*GFP-LC3B embryos also show an increased number of GFP-LC3B positive structures (Fig. 2I-J). Interestingly, the average size of these structures was significantly reduced (Fig. 2I, K). Moreover, FACS-based quantification [17] showed that GFP-LC3B degradation in response to starvation in *Atg4d*^−/−^ cells was comparable to that observed in the corresponding *WT* controls (Fig. 2L, M). Accordingly, analysis of autophagic degradation of radio-labelled proteins [18] showed comparable levels of autophagy-dependent proteolysis upon nutrient deprivation between *WT* and *Atg4d*-deficient cells (Fig. 2N and Fig. S4). Furthermore, the observed alterations in *Atg4d*^−/−^ cells cannot be explained by an up-regulation in the activity of the main autophagy-initiating complexes (Fig. 2O-Q).

### Increased autophagosome content in the absence of ATG4D

**Fig. 3:**
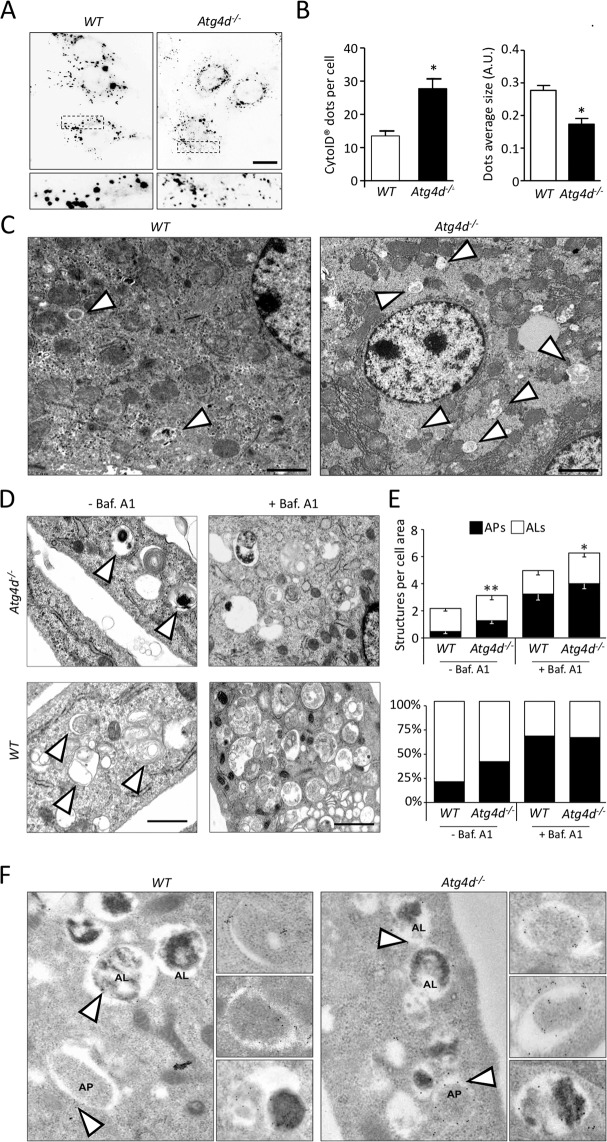
Autophagosome cellular content in the absence of ATG4D. **A** Representative images of Cyto-ID®-labelled autophagosomes in *WT* and *Atg4d*^*−/−*^ MEFs upon 2 h of Torin1 treatment as an autophagy inducer. Similar results were obtained upon nutrient deprivation. **B** Quantification of the data from (**A**). **C** Representative TEM images of liver tissue samples (hepatocytes) from *WT* and *Atg4d*-deficient mice fed ad libitum. Autophagic structures are labelled by arrowheads. **D** Representative TEM images from *WT* and *Atg4d*^*−/−*^ MEFs cultured upon 4 h of nutrient deprivation, either in the presence or absence of BafA1. Arrowheads label autophagic structures (except in the samples treated with BafA1, for clarity purposes). **E** Quantification of autophagosome content (AP, autophagosome; AL, autolysosome) in *WT* and *Atg4d*-deficient MEFs in the indicated conditions in (**D**). **F** Representative immunogold TEM images against GFP in *WT* and *Atg4d*^*−/−*^ MEFs expressing the GFP-LC3B transgene. Insets show GFP-LC3B labelling of nascent (up), early (middle) or late (bottom) autophagosomes. Bars represent mean ± SEM (**B**) or mean ± SD (**E**) (*N* > 80 cells per condition in (**B**); *N* > 250 cells in (**E**)). Scale bars: 10 µm (**A**) and 2 µm (**C**, **D**). *P < 0.05, 2-tailed unpaired Student’s *t* test.

In yeast, Atg8 may be inappropriately conjugated to non-autophagosomal membranes [19]. Thus, we decided to extend our analyses to mATG8s-independent autophagosome markers. As shown in Fig. 2P-Q, *Atg4d*^*−/−*^ MEFs showed an increased number of syntaxin 17 (STX17TM)-positive structures, which correspond to successfully-formed autophagosomes [20, 21]. STX17 mediates autophagosome-lysosome fusion through its interaction with VAMP8 at the lysosomal/endolysosomal membrane [21]. Consistently, *Atg4d*^*−/−*^ MEFs also showed a higher number of VAMP8 and lysotracker-positive vesicles (Fig. 2P-Q). In this sense, staining with the commercial probe Cyto-ID^®^, which labels autophagosomes independently of mATG8 proteins, confirmed the increase in the number of starvation-induced Cyto-ID^®^ positive structures, which also showed a significantly reduced average size in *Atg4d*^*−/−*^ MEFs (Fig. 3A-B). Consistently, transmission electron microscope (TEM) analyses revealed an increased number of autophagic structures in tissue and MEFs samples from *Atg4d*^*−/−*^ mice, either in the presence or absence of BafA1 (Fig. 3C-E). Moreover, immunogold staining against GFP in MEFs stably expressing GFP-LC3B revealed that it was exclusively associated with autophagic structures (nascent, early, or late autophagosomes) both in *WT* and *Atg4d*^*−/−*^ MEFs (Fig. 3F). Together, these results rule out the possibility that the increase in the lipidated pool of mATG8s in the absence of ATG4D derives from inappropriate conjugation to non-autophagic membranes.

### Deletion of *Atg4d* only leads to accumulation of lipidated mATG8s

**Fig. 4:**
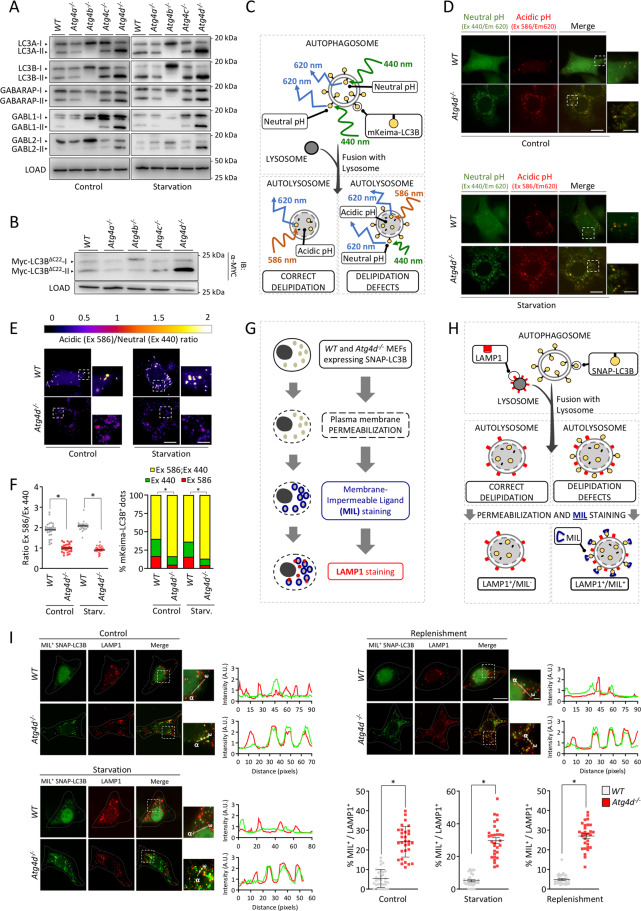
*Atg4d* disruption leads to defects in delipidation of mammalian ATG8 proteins. **A** Immunoblotting analyses showing lipidation status of all murine ATG8 proteins in MEFs deficient for each of the ATG4 members, either in full media (left) or upon 4 h of nutrient deprivation (right). **B** Immunoblotting analyses against Myc epitope in MEFs deficient for each ATG4 member, stably-expressing a version of Myc-tagged LC3B exposing the reactive glycine at its carboxyl end amino-acid (LC3B∆C22). **C** Schematic representation of the different possible autophagosome-related structures containing mKeima-LC3B. **D** Representative images for *WT* and *Atg4d*^−/−^ MEFs stably expressing Keima-LC3B, cultured in the indicated conditions. **E** Representative images showing the fluorescence intensity ratio from both mKeima-LC3B signals (Ex586-Em620)/(Ex440-Em620). **F** Left, quantification of mKeima-LC3B ratio from (Ex586-Em620)/(Ex440-Em620) signals. Each point value represents the average ratio of mKeima-LC3B positive structures of a single cell. Right, relative abundance of mKeima-LC3B positive structures in both *WT* and *Atg4d*^−/−^ MEFs in the indicated culture conditions. **G** Schematic strategy of the SNAPtag®-LC3B assay developed to specifically monitor LC3B present at the cytosolic leaflet of the autolysosomal membrane. **H** Schematic representation of the expected results for either normal or defective LC3 delipidation. **I** Representative pictures and quantification of *WT* and *Atg4d*^−/−^ MEFs stably expressing SNAPtag®-LC3B and double-stained with MIL (green) and Alexa594®-conjugated anti-LAMP1 antibody (red) in the indicated conditions. Graphs show intensity profiles for fluorescent signals along the direction indicated in the insets (α−ω). Scale bars 10 μm. The percentage of LAMP1-positive dots which are also positive for MIL labelling is shown. LOAD: β-actin. Scale bars: 10 μm, 3 μm (**E**) and 2 μm (**I**) in insets. Measurements were done with 30 cells per genotype and treatment. *P < 0.05, 2-tailed unpaired Student’s *t* test.

Our previous results suggest that ATG4D might be the main in vivo delipidating enzyme for mATG8 proteins, which would accumulate bound to autophagic membranes in the absence of ATG4D. Thus, we compared the status of the different mATG8s in MEFs deficient for each of the mammalian ATG4s. To that purpose, we extracted MEFs from *Atg4b-* and *Atg4c*-deficient mice [6] and generated *Atg4a*-deficient MEFs by using the CRISPR/Cas9 system (Fig. S5A). Immunoblotting analyses revealed that only ATG4D deficiency leads to a substantial increase in the levels of lipidated mATG8s, either under control or nutrient-free conditions (Fig. 4A). *Atg4d*^*−/−*^ MEFs do not show any defect in the initial cleavage of pro-mATG8 proteins (Fig. S5B-F), contrasting with *Atg4b*^*−/−*^ cells that show accumulation of uncleaved pro-mATG8s, which cannot bind to autophagosomal membranes (Fig. S5B-F), precluding the analysis of ATG4B delipidating activity. Thus, we generated MEFs lines stably expressing a C-terminal deletion mutant of LC3B (Myc-LC3B^ΔC22^), which bypasses the initial proteolytic cleavage of pro-LC3, being directly incorporated into autophagic membranes [22]. As shown in Fig. 4B, analysis of Myc-LC3B^ΔC22^ lipidation in MEFs deficient for each ATG4 proteases confirmed that only *Atg4d* deletion leads to increased lipidation of LC3B, supporting a principal role for ATG4D protease in the delipidation of mATG8s.

### Loss of ATG4D leads to defects in mATG8s delipidation

Delipidation of ATG8 proteins has been documented in cell-free systems [23, 24]. However, to our knowledge, no information on ATG8s delipidation in mammalian living cells has been reported so far.

Thus, we developed different experimental strategies to monitor the delipidation of mATG8s in living cells. We hypothesized that a delipidation defect would lead to an increased presence of mATG8 proteins at the cytosolic leaflet of the autolysosomal membrane after the autophagosome-lysosome fusion. In order to test this hypothesis, we took advantage of the properties of mKeima, which shows a dual excitation/emission pattern for neutral and acidic pH [25]. If fused with a mATG8 protein, this unique property would allow us to monitor its relative abundance between the lumen (acidic pH) and the cytosolic leaflet of the autolysosomal membrane (neutral pH). In the case of a delipidation defect, an increased presence of mKeima-mATG8 at the cytosolic leaflet of the autolysosomal membrane would lead to a higher relative fluorescence associated to cytosolic neutral pH in autophagic structures (Fig. 4C). Thus, we generated *WT* and *Atg4d*^−/−^ cells stably expressing mKeima-LC3B and analyzed the distribution of both acidic and neutral pH-associated signals in mKeima-LC3B puncta. In all our experimental settings, the fluorescence intensity ratio between acidic and neutral pH-associated signals (Ex586/Ex440) was very close to 2 in *WT* cells, suggesting that the majority of mKeima-LC3B molecules were present in the lumen of acidic vesicles (Fig. 4D-F). By contrast, this ratio was very close to 1 in *Atg4d*^−/−^ cells, which suggests an even distribution of mKeima-LC3B between the lumen and the cytosolic membrane leaflet of these acidic vesicles (presumably autolysosomes). Notably, *Atg4d* KO cells do not show any increase in lysosomal pH, which would also explain the observed results (Fig. S5G-H).

In addition, we decided to complement these studies by specifically monitoring the pool of lipidated LC3B attached to the cytosolic leaflet of the autolysosomal membrane. To do so, we generated *WT* and *Atg4d*^*−/−*^ MEFs stably-expressing LC3B fused to a SNAP-tag^®^ domain, which allows fluorescent labeling of proteins in a time-controlled fashion [26]. Next, we adapted a recently published protocol [27] for the specific detection of lipidated LC3B at the cytosolic leaflet of the external autophagosomal/autolysosomal membranes (Fig. 4G). This protocol is based on the specific permeabilization of the plasma membrane (without permeabilization of other cellular membranes) to release cytosolic SNAP-LC3B-I and sequential labeling with a fluorescent Membrane Impermeable SNAP-Surface^®^ Ligand (MIL). This ligand will label the accessible pool of SNAP-LC3B-II. Thus, SNAP-LC3B/MIL^+^, LAMP-1^+^ double-positive structures will represent autolysosomes showing lipidated SNAP-LC3B-II at their cytosolic membrane leaflet (Fig. 4H). As shown in Fig. 4I, this assay revealed a significant increase of these SNAP-LC3B/MIL^+^, LAMP-1^+^ double-positive structures in *Atg4d*^*−/−*^ cells in different experimental conditions (Fig. 4I), in line with our previous results pointing to a delipidation defect in the absence of ATG4D (Fig. 4I).

Together, these results show that the accumulation of the membrane-bound forms of LC3s and GABARAPs in the absence of ATG4D stems from a major role for ATG4D in their delipidation.

### *Atg4d*^*−/−*^ mice show age-dependent cerebellar neurodegeneration and motor dysfunction

**Fig. 5:**
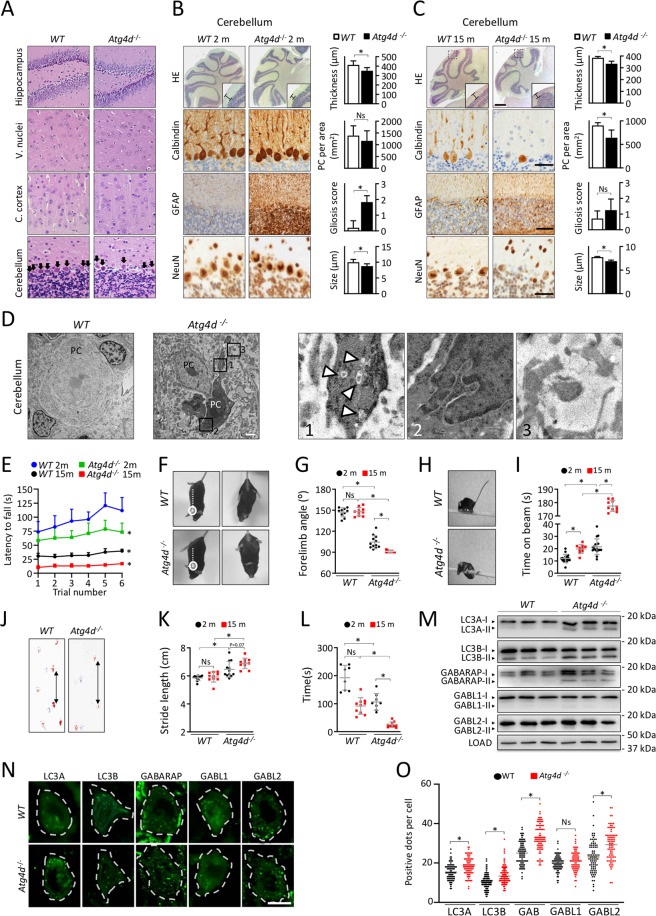
Age-associated cerebellar neurodegeneration in *Atg4d*^*−/−*^ mice. **A** Representative H&E images from 15-month-old *WT* and *Atg4d*^*−/−*^ mice hippocampus, vestibular nuclei, cerebral cortex, and cerebellum. Arrowheads label Purkinje cells (PC) bodies. Scale bars: 50 μm. **B**–**C** Representative images and quantification of histological analysis in 2- (**B**) and 15- (**C**) month-old *WT* and *Atg4d*^*−/−*^ mice cerebella. Up, representative H&E images. Scale bar 400 μm. Bars label gray matter thickness. Middle up, representative images for calbindin IHC. Scale bar: 20 μm. Middle bottom, IHC against Glial Fibrillary Acidic Protein (GFAP). Scale bar: 40 μm. Bottom, IHC against Neuronal Nuclei (NeuN). Scale bar: 20 μm. **D** Representative transmission electron microscopy (TEM) images showing the ultrastructure of *WT* and *Atg4d*^*−/−*^ mice cerebellar PCs. Scale bar, 1 μm. Autophagosomes (1), abnormal nuclear envelope invaginations (2) and swollen radial processes (3) in *Atg4d*^*−/−*^ PCs are shown in insets. **E** Results from Rotarod analyses of 2- and 15-month-old *WT* and *Atg4d*^*−/−*^ mice. Asterisks show significant differences when areas under the curve are compared to 2-month-old *WT* group. **F** Representative limb postures responding to tail suspension in 15-month-old *WT* and *Atg4d*^*−/−*^ mice. **G** Quantification of forelimb angles against body axes in the tail suspension test. **H** Representative images for raised-beam test in 15-month-old *WT* and *Atg4d*^*−/−*^ mice. **I** Performance on the raised-beam test of 2- and 15-month-old mice measured by the time mice spend before reaching the end of the beam. **J** Representative paw placement records of 15-month-old *WT* and *Atg4d*^*−/−*^ mice after footprint pattern analysis. **K** Quantification of stride length of *WT* and *Atg4d*^*−/−*^ mice. Data are presented as mean and SD of at least 5 mice per group. **L** Results from grip strength analyses of 2- and 15-month-old *WT* and *Atg4d*^*−/−*^ mice. Graph represents the time mice were able to hold themselves to a grid when inverted in the inverted-cling grip test. **M** Representative immunoblots of endogenous ATG8-like proteins in the cerebellum from *WT* and *Atg4d*^*−/−*^ mice. **N** Representative images of immunofluorescence analysis of endogenous mATG8 proteins in *WT* and *Atg4d*^*−/−*^ cerebellar PCs. **O** Quantification of the data shown in (**N**). Bars represent mean ± SD. *N* = 6, 2-month-old mice per genotype (**B**). *N* = 7, 15-month-old mice per genotype (**C**); *N* > 11, 2-month-old mice and *N* = 10, 15-month-old mice per genotype (**E**, **G**); *N* = 17, 2-month-old mice and *N* = 10, 15-month-old mice per genotype (**I**); *N* = 12, 2-month-old mice and *N* = 10, 15-month-old mice per genotype (**K**); *N* = 8, 2-month-old mice and *N* = 10, 15-month-old mice per genotype (**L**); *N* = 4 mice per genotype (**M**); *N* > 85 cells per condition (b). *P < 0.05, 2-tailed unpaired Student’s *t* test (**B**, **C**, and **O**) and two-way ANOVA followed by Dunnett´s post hoc test (**E**, **G**, **I**, **K** and **L**).

Although *Atg4d* deletion leads to noticeable alterations at the molecular level, we did not observe any obvious phenotype in *Atg4d*^*−/−*^ mice during approximately their first year of age. Accordingly, histological analyses in different tissues (such as heart, liver, kidneys or skeletal muscle among others) did not reveal any observable differences between *WT* and *KO*-derived samples (Fig. S5I). However, we could detect a significantly lower number of Purkinje cells (PCs) in cerebellar sections from aged *Atg4d*^−/−^ mice (Fig. 5A-C). Moreover, PC-specific calbindin immunohistochemistry staining revealed alterations in PC cell body alignment (Fig. S6A), a feature associated with cerebellar ataxia [28]. Moreover, a reduced cell body size and an abnormal nuclear shape were observed in *Atg4d*^−/−^ Purkinje neurons upon anti-NeuN staining (Fig. 5B-C). As shown in Fig. 5B-C, a reduction in the thickness of the cerebellar molecular layer and an increase in GFAP staining, which usually indicates reactive gliosis in response to neuronal damage and/or neuroinflammation, were also observed [29]. These features, already observed in 2-months old *Atg4d*^−/−^ mice cerebellum persisted in 15-months old mice, in which a significant PC loss could be observed (Fig. 5C). Remarkably, all these alterations were neither associated with any increase in Ubiquitin-positive protein aggregates nor by an increase in dUTP nick end labeling upon TUNEL analysis (Fig. S6B-C). As shown in Fig. 5D, TEM analyses revealed that samples from *Atg4d*^−/−^ presented a disorganized ultrastructure, with PCs surrounded by numerous electron-transparent gaps, which likely correspond to swollen radial processes from glial cells [30]. PCs were smaller and shrunken with their nuclei often showing occasional invaginations and granular appearance in *Atg4d*^−/−^ mice samples (Fig. 5D). Additionally, *Atg4d*^−/−^ PCs showed a high number of autophagosomes with cytoplasmic cargo and increased electron density in their cytoplasm (Fig. 5D). Most of these ultrastructural abnormalities in *Atg4d*^−/−^ PCs resemble alterations documented in *dark cell degeneration*, a type of PC demise usually linked to excitotoxicity [31, 32]. In order to see if these cerebellar alterations are functionally associated with motor coordination defects, we subjected 2- and 15-month-old, age-matched *WT* and *Atg4d*^−/−^ mice to a set of functional tests to characterize features associated with cerebellar function. Despite not showing any observable difference when unchallenged, knockout mice showed reduced performance compared to *WTs* in Rotarod tests, as they took significantly less time before falling (Fig. 5E). In the tail suspension test, *Atg4d-*deficient mice showed a significant difference in the forelimb angles against body axe (Fig. 5F, G). Similarly, the performance of null mice in raised-beam balance tests was poorer than *WT* controls (Fig. 5H, I and S8A). Foot-printing analyses revealed a significant gait alteration in mutant mice characterized by an increase in stride length (Fig. 5J, K). Finally, grip-strength experiments (inverted-cling grip test) also showed a reduced performance of mutant mice (Fig. 5L). All these alterations, which were not sex-biased in any of the performed tests (Fig. S7), became more pronounced in aged *Atg4d*^−/−^ mice, indicating that ATG4D loss leads to progressive cerebellar ataxia, in line with our immuno-histological analyses showing progressive PC demise. In addition, mutant mice also performed worse than their age-matched counterparts in functional tests associated to the neurological function of other parts of the CNS, such as open-field and LTM recognition tests (Fig. S8). This suggests that although cerebellar function is the most severely affected by neurodegeneration, ATG4D loss leads to alterations in the function of other parts of the CNS.

### Motor dysfunction in *Atg4d*^*−/−*^ mice is reverted by modulating GABA_A_ receptors activity

**Fig. 6:**
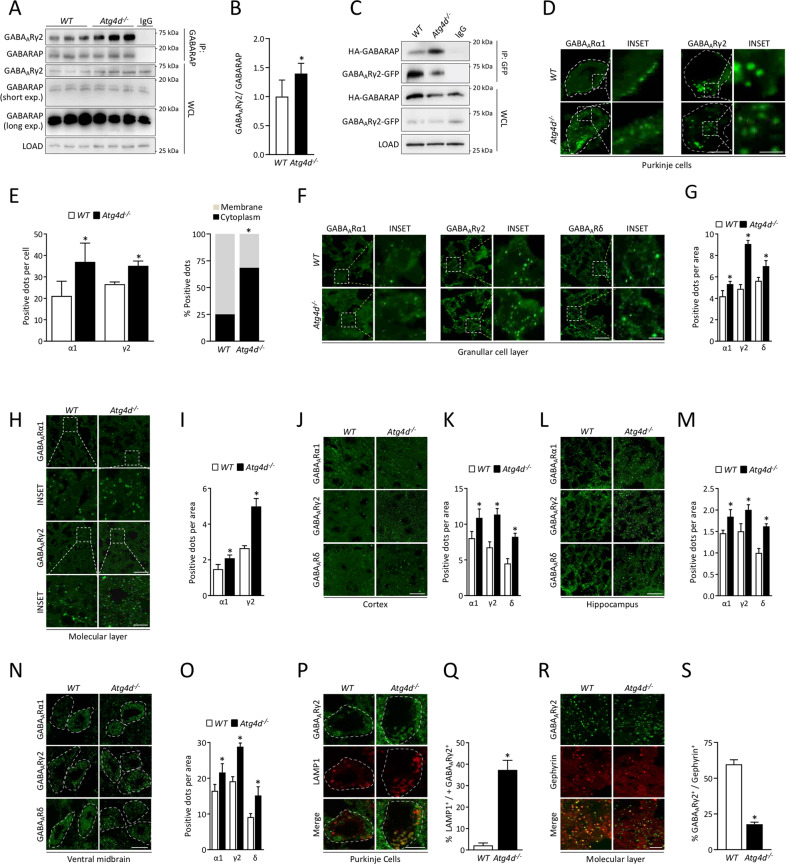
Analysis of GABA_A_R subunits subcellular localization. **A** Representative immunoblots for co-immunoprecipitation of GABA_A_Rγ2 with GABARAP in cerebella from *WT* and *Atg4d*^*−/−*^ mice. **B** Quantification of the data shown in (**A**). For (**A**), tissue extract from a *WT* mouse was used as a control for IgG immunoprecipitation. **C** Representative immunoblots for co-immunoprecipitation of GABA_A_Rγ2 with GABARAP in *WT* and *Atg4d*^*−/−*^ HEK293T cells overexpressing GABA_A_Rγ2-GFP and HA-GABARAP. Transfected *WT* HEK293T cells were used as control for IgG immunoprecipitation. **D** Immunofluorescence analyses of GABA_A_Rα1 and GABA_A_Rγ2 in *WT* and *Atg4d*^*−/−*^ PCs. **E** Quantification of the data shown in (**D**). **F** Immunofluorescence analyses of GABA_A_Rα1, GABA_A_Rγ2, and GABA_A_Rδ in the granular cell layer from *WT* and *Atg4d*^*−/−*^ cerebellar cortex. **G** Quantification of the data shown in (**F**). **H** Immunofluorescence analyses of GABA_A_Rα1 and GABA_A_Rγ2 in *WT* and *Atg4d*^*−/−*^ molecular layer of the cerebellar cortex. **I** Quantification of the data shown in (**H**). **J** Immunofluorescence analyses of GABA_A_Rα1, GABA_A_Rγ2, and GABA_A_Rδ in cerebral cortex from *WT* and *Atg4d*^*−/−*^. **K** Quantification of the data shown in (**J**). **L** Immunofluorescence analyses of GABA_A_Rα1, GABA_A_Rγ2, and GABA_A_Rδ in hippocampus from *WT* and *Atg4d*^*−/−*^. **M** Quantification of the data shown in (**L**). **N** Immunofluorescence analyses of GABA_A_Rα1, GABA_A_Rγ2, and GABA_A_Rδ in ventral midbrain from *WT* and *Atg4d*^*−/−*^. **O** Quantification of the data shown in (**N**). **P** Immunofluorescence analyses of GABA_A_Rγ2 and LAMP1 in *WT* and *Atg4d*^*−/−*^ PCs. **Q** Quantification of the data shown in (**P**). Colocalization of GABA_A_Rγ2 and LAMP1 is calculated as the percentage of GABA_A_Rγ2/LAMP1 double-positive structures divided by the total number of GABA_A_Rγ2 positive structures per cell area. **R** Immunofluorescence analyses of GABA_A_Rγ2, and gephyrin in *WT* and *Atg4d*^*−/−*^ cerebellar cortex molecular layer. **S** Quantification of the data shown in (**R**). Colocalization of GABA_A_Rγ2 and gephyrin is calculated as the percentage of GABA_A_Rγ2/gephyrin double-positive structures divided by the total number of gephyrin positive structures per cell area. LOAD: GAPDH, WCL: Whole cell lysate. Bars represent mean ± SD (*N* > 85 cells per mouse, 3 mice per genotype (**D**, **F**, **H**, **J**, **L**, **N**, **P** and **R**). Scale bars: 10 μm, insets 1 μm. ^*^P < 0.05, 2-tailed unpaired Student’s *t* test. In (**B**), bars represent mean ± SD (*N* = 7 *WT* mice and 6 *Atg4d*^*−/−*^ mice).

Consistent with our results in other tissues, we detected an increased presence of lipidated forms and positive structures of mATG8s in different areas of the CNS, including the cerebellum (Fig. 5M-O, Fig. S9). Apart from their functions in autophagic degradation, GABARAP plays a role in GABA receptor trafficking. In fact, GABARAP binds to the intracellular loop of the γ2 subunit of the GABA_A_ receptor [33, 34]. As shown in Fig. 6A-C, we could detect a significant increase in the interaction between these two proteins in homogenates from *Atg4d*^*−/−*^ mice cerebella and also in *Atg4d*^*−/−*^ MEFs, as measured by immunoprecipitation experiments. As GABARAP interaction with GABA_A_Rγ_2_ has been reported to promote GABA_A_R transport to the plasma membrane in neurons [35], we decided to analyze the subcellular distribution of different GABA_A_ receptors subunits in different parts of the CNS in WT and *Atg4d*^*−/−*^ mice. As shown in Fig. 6D-I, we detected a higher number of positive structures for GABA_A_Rα_1_ and GABA_A_Rγ_2_, in PCs, granular cells and also in the molecular layer of mutant mice cerebella. Moreover, the number of positive structures for GABA_A_Rδ subunit was significantly higher inside granular cells cytoplasm (Fig. 6F, G). Similarly, the number of GABA_A_Rα_1_, GABA_A_Rγ_2_ and GABA_A_Rδ subunits was also significantly higher in different parts of *Atg4d*^*−/−*^ mice CNS, such as cerebral cortex (Fig. 6J, K); hippocampus (Fig. 6L, M) and ventral midbrain (Fig 6 N, O). Interestingly, *Atg4d*^*−/−*^ neurons, including PCs, showed a reduced localization of GABA_A_ receptors at the plasma membrane, as revealed by immunofluorescence against GABA_A_Rα_1_-subunit (Fig. 6D, E). Moreover, we detected a significant colocalization of GABA_A_Rγ_2_-subunit positive structures and the lysosomal marker LAMP-1 in *Atg4d*^*−/−*^ PCs, which was barely detected in WT cells (Fig. 6P, Q). This may suggest that in the absence of ATG4D, GABA_A_R receptors fail to be correctly transported to the plasma membrane and are targeted to lysosomal compartments. Consistently, the number of GABA_A_R synaptic clusters (as measured by gephyrin and GABA_A_Rγ_2_ colocalization) was significantly reduced in the molecular layer of *Atg4d*^*−/−*^ mice cerebella (Fig. 6R, S).

**Fig. 7:**
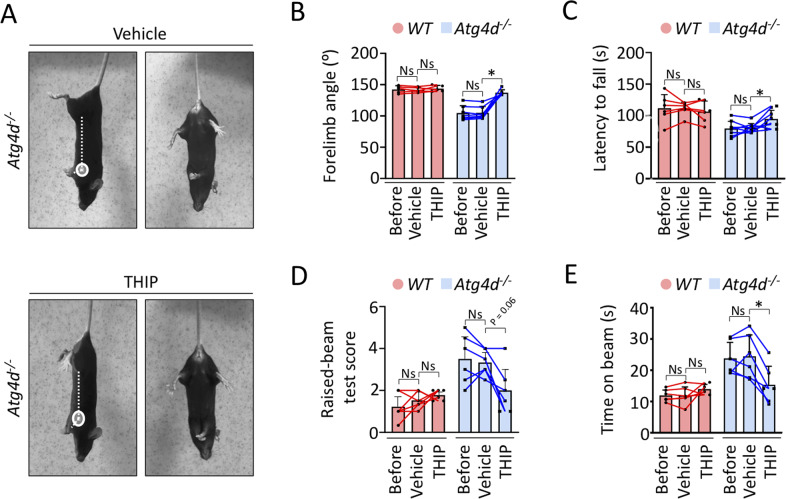
Effects of THIP administration in *Atg4d*^*−/−*^ mice. **A**–**E** Effects of THIP administration in tail suspension test (**A**, **B**), Rotarod (**C**) and raised-beam test (**D**, **E**) analyses in 2-month-old *WT* and *Atg4d*^*−/−*^ mice. Bars represent mean ± SD. ^*^P < 0.05, repeated measures ANOVA followed by Dunnett´s post hoc test. *N* > 6 mice per genotype.

These results point to defects either in their trafficking or clustering in the absence of ATG4D. Thus, we decided to evaluate the effects of different GABA receptors modulators, such as the GABA_A_ receptors agonists muscimol, THIP and baclofen, as well as the GABA_A_ receptor antagonist bicuculline in young *Atg4d*^*−/−*^ mice, which do not yet manifest cerebellar PCs loss. As shown in Fig. S10, neither muscimol, nor baclofen, nor bicuculline had a significant effect in the performance of mutant mice. However, treatment with THIP was able to significantly improve *Atg4d*^*−/−*^ mice performance, without exerting any noticeable effect in WT mice (Fig. 7A-E). THIP is a selective GABA receptors agonist, which mainly acts on extrasynaptic GABA_A_ receptors. These receptors enable neurons to sense low ambient GABA concentrations present in the extracellular space in order to generate a form of tonic inhibition, which reduces the excitability of cerebellar granule cells and thus PCs firing rate [36–38]. The fact that THIP treatment significantly ameliorates motor coordination in young *Atg4d*^*−/−*^ mice, which still preserve cerebellar PC layer, suggests that the observed cerebellar dysfunction upon ATG4D loss is a result of GABA_A_ receptor alterations.

### Human *ATG4D* allelic variants are associated with neurodegeneration

**Fig. 8:**
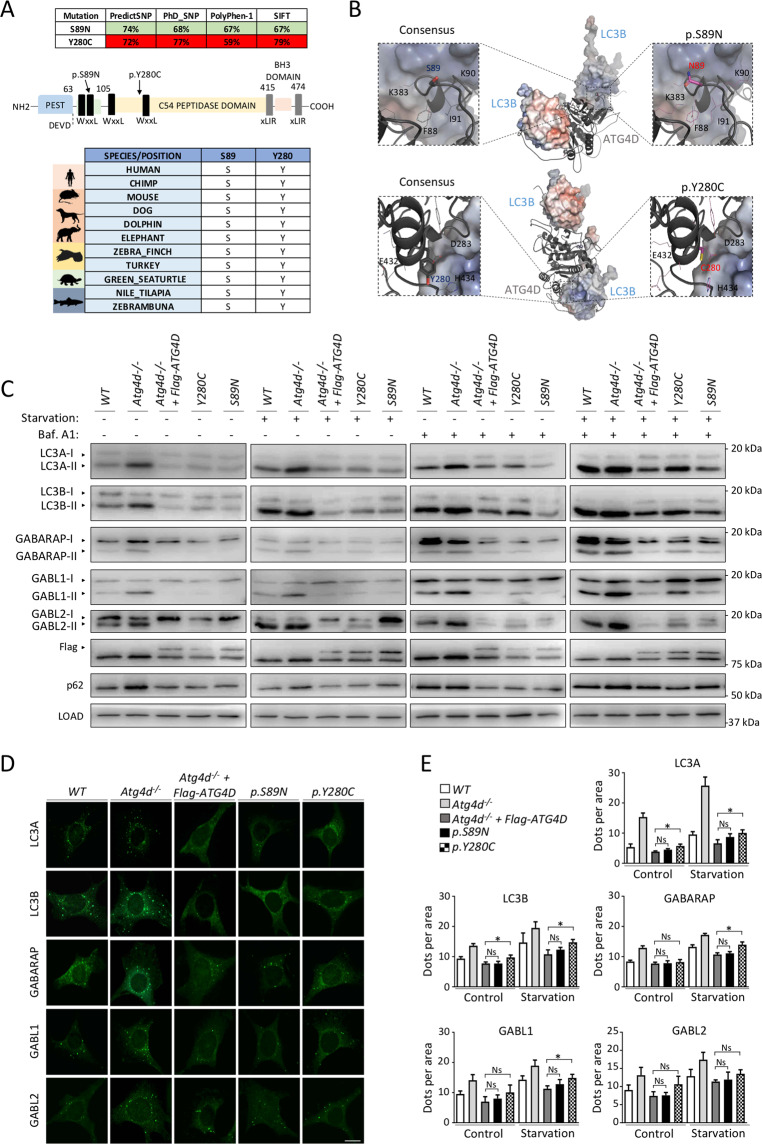
Effects of *HsATG4D* p.S89N and p.Y280C allelic variants. **A** Up, predicted impact of p.S89N and p.Y280C variants using publicly available prediction algorithms. Numbers indicate the probability of a determined variant to be neutral (green boxes) or detrimental (red boxes) for protein structure stability. Middle, schematic representation of the different protein domains present in ATG4D. Bottom, scheme showing conservation of S89 and Y280 residues across evolutionarily-distant vertebrate species. **B** Computer-based model of HsATG4D 3D structure interacting with a substrate and an auxiliary LC3B. The position of p.S89N and p.Y280C variants and adjacent residues are shown in more detail. **C** Immunoblotting analyses against mATG8 proteins in WT, *Atg4d*^*−/−*^ MEFs and *Atg4d*^*−/−*^ MEFs stably expressing either Flag-HsATG4D, Flag-HsATG4D p.S89N or Flag-HsATG4D p.Y280C in the indicated conditions. **D** Representative images of immunofluorescence against endogenous mATG8 proteins in WT, *Atg4d*^*−/−*^ MEFs and *Atg4d*^*−/−*^ MEFs stably expressing either Flag-HsATG4D, Flag-HsATG4D p.S89N or Flag-HsATG4D p.Y280C in the indicated conditions. Images show cells cultured in full medium. **E** Quantification of the data from (**D**). LOAD: β-actin. Bars represent mean ± SD (*N* > 80 cells per condition). Scale bar: 10 μm. ^*^P < 0.05, 2-tailed unpaired Student’s *t* test.

During the course of our analyses, we could find a publicly accessible blog relating the case of a young patient suffering from a rare form of cerebellar ataxia associated to the presence of two different *ATG4D* gene variants (http://neurodegenerativeatg4d.blogspot.com). The association of the patient’s phenotype with *ATG4D* c.266 G > A, pSer89Asn (paternally-inherited) and c.839 A > G, pTyr280Cys (maternally-inherited) variants was confirmed through personal communication with the patient’s family. Interestingly, both Ser89 and Tyr280 are conserved through evolution in all analyzed species (Fig. 8A). Pathogenicity estimates of these *ATG4D* variants by different independent algorithms predicted Tyr280Cys to likely disrupt protein stability and pSer89Asn to be neutral (Fig. 8A). Interestingly, these residues are located at opposite sides of the ATG4D predicted 3D structure, based on available ATG4A (PDB entry 2P82) and ATG4B [39] 3D structures (Fig. 8B). In mammalian ATG4 proteins, two molecules (a “substrate mATG8” and a “non-substrate mATG8”) interact with the inactive protease and trigger conformational changes that expose the catalytic pocket [39]. In this regard, pTyr280Cys is located near to the interacting region with the substrate LC3B molecule, close to its Ser90 and Lys65 residues, whereas pSer89Asn is close to Arg69 and Lys65 of the non-substrate LC3B molecule.

As shown in Figs. 8C-E and S11, both variants were able to partially revert the increase in mATG8s lipidation caused by ATG4D loss. However, when compared to the effect of the consensus HsATG4D form, the ability of these variants to reduce either puncta or lipidation of mATG8s was lower in most cases, suggesting that these mutations compromise ATG4D function (Fig. 8A, C-E). Thus, the combination of these mutations, interfering with both mATG8s docking sites of ATG4D at the same time, may further hamper in vivo ATG4D activity.

Taken together, our results show that ATG4D is the main mATG8 delipidating enzyme in mammalian cells and plays an evolutionarily conserved role against neurodegeneration, as reported for other species [40].

## Discussion

In this work, we have shown that ATG4D is the main delipidating enzyme for mammalian mATG8s, which accumulate in their membrane-bound form in the absence of this protease. Previous works had shown that ATG4D protease was able to delipidate GABARAPL1 protein in in vitro cell-free experiments upon proteolytic activation by caspase-3 [8]. However, a role of ATG4D as a delipidating enzyme toward the rest of mATG8 proteins in vivo had not been previously addressed. Interestingly, ATG4D deficiency affects also autophagosome number and size without significantly altering autophagic flux. We have shown that the increased presence of membrane-bound forms of mATG8s derives from defects in their delipidation. It is particularly interesting that the non-delipidated pool of mATG8 proteins remains at the cytosolic leaflet of the autolysosomal membrane. This suggests that delipidation of mATG8 proteins is not a necessary event for autophagosome/lysosome fusion, which can efficiently occur in the absence of delipidation. In mice, the main physiological impact of *Atg4d* disruption is cerebellar neurodegeneration. Functionally, *Atg4d*^−/−^ mice show signs of cerebellar ataxia, such as motor coordination defects and gait abnormalities, whose severity increases with age. These features are associated to alterations in the trafficking/clustering of different GABA_A_ receptors subunits, likely as a result of the altered interaction between GABARAP and GABA_A_ γ2-subunit, in the absence of ATG4D.

One possibility is that due to the retention of GABARAP proteins on autophagic structures, and given that the interaction between GABARAP and GABA_A_ γ2-subunit is increased in the absence of ATG4D, a significant proportion of GABA_A_ receptors might be degraded through autophagy, thus explaining the observed reduced localization of these receptors at the plasma membrane and at synaptic clusters. However, given that a great number of cellular factors are involved in GABA receptors trafficking and clustering, other different alternative mechanisms that could explain our observations cannot be ruled out. Despite these considerations, it is remarkable that treatment with the GABA receptor agonist THIP is able to revert the motor coordination defects of young *Atg4d*^−/−^ mice. This demonstrates that defects in GABA signaling are already present before the onset of neurodegeneration in *Atg4d*^−/−^ mice. In this sense, it is intriguing that while PCs progressively decline, other neuronal types remain preserved upon ATG4D deficiency. PCs are the only output neurons of the cerebellar cortex and receive excitatory inputs from climbing and parallel fibers and inhibitory inputs from basket, stellate and neighboring PCs [41]. Thus, it is possible that the persistent autophagy/GABA-receptors alterations observed in *Atg4d*-deficient cells would sensitize neurons to inadequate excitatory/inhibitory input balance, ultimately resulting in the observed loss of PCs, particularly vulnerable to excitotoxicity [42]. In fact, the ultrastructural abnormalities observed in *Atg4d*^−/−^ PCs correspond to alterations documented in *dark cell degeneration*, a type of PC demise usually linked to excitotoxicity [31, 32], which supports this idea.

In summary, our results show that ATG4D is the principal ATG8 delipidating enzyme in mammalian cells. Loss of ATG4D leads to an increased cellular stationary content of autophagosomes and autophagy-related structures without compromising autophagy flux. In mice, these molecular alterations lead to alterations in GABA_A_ receptors trafficking and targeting, which alter cerebellar function, progressively leading to neurodegeneration and to the development of defects in motor coordination. Further studies will be required to fully characterize the fine mechanisms linking the molecular alterations caused by ATG4D loss to the development of neurodegenerative features.

## Supplementary information

Supplemental Figure legends

Supplemental Figure 1

Supplemental Figure 2

Supplemental Figure 3

Supplemental Figure 4

Supplemental Figure 5

Supplemental Figure 6

Supplemental Figure 7

Supplemental Figure 8

Supplemental Figure 9

Supplemental Figure 10

Supplemental Figure 11

## References

[1] Marino G, Lopez-Otin C (2004). Autophagy: molecular mechanisms, physiological functions and relevance in human pathology. Cell Mol Life Sci.

[2] Klionsky DJ, Abdelmohsen K, Abe A, Abedin MJ, Abeliovich H, Acevedo Arozena A (2016). Guidelines for the use and interpretation of assays for monitoring autophagy (3rd edition). Autophagy.

[3] Ichimura Y, Kirisako T, Takao T, Satomi Y, Shimonishi Y, Ishihara N (2000). A ubiquitin-like system mediates protein lipidation. Nature.

[4] Fernandez AF, Lopez-Otin C (2015). The functional and pathologic relevance of autophagy proteases. J Clin Investig.

[5] Shpilka T, Weidberg H, Pietrokovski S, Elazar Z (2011). Atg8: an autophagy-related ubiquitin-like protein family. Genome Biol.

[6] Marino G, Fernandez AF, Cabrera S, Lundberg YW, Cabanillas R, Rodriguez F (2010). Autophagy is essential for mouse sense of balance. J Clin Investig.

[7] Nguyen N, Olivas TJ, Mires A, Jin J, Yu S, Luan L (2020). The insufficiency of ATG4A in macroautophagy. J Biol Chem.

[8] Betin VM, Lane JD (2009). Caspase cleavage of Atg4D stimulates GABARAP-L1 processing and triggers mitochondrial targeting and apoptosis. J Cell Sci.

[9] Subramani S, Malhotra V (2013). Non-autophagic roles of autophagy-related proteins. EMBO Rep..

[10] Hevers W, Luddens H (1998). The diversity of GABAA receptors. Pharmacological and electrophysiological properties of GABAA channel subtypes. Mol Neurobiol.

[11] Cossart R, Bernard C, Ben-Ari Y (2005). Multiple facets of GABAergic neurons and synapses: multiple fates of GABA signalling in epilepsies. Trends Neurosci.

[12] Ben-Ari Y, Khazipov R, Leinekugel X, Caillard O, Gaiarsa JL (1997). GABAA, NMDA and AMPA receptors: a developmentally regulated ‘menage a trois’. Trends Neurosci.

[13] Reed MG, Howard CV (2010). GSDEY One-stop stereology: the estimation of 3D parameters using isotropic rulers. J Microsc.

[14] Codina-Martinez H, Fernandez-Garcia B, Diez-Planelles C, Fernandez AF, Higarza SG, Fernandez-Sanjurjo M (2020). Autophagy is required for performance adaptive response to resistance training and exercise-induced adult neurogenesis. Scand J Med Sci Sports.

[15] Chomczynski P, Sacchi N (2006). The single-step method of RNA isolation by acid guanidinium thiocyanate-phenol-chloroform extraction: twenty-something years on. Nat Protoc.

[16] Mizushima N, Yamamoto A, Matsui M, Yoshimori T, Ohsumi Y (2004). In vivo analysis of autophagy in response to nutrient starvation using transgenic mice expressing a fluorescent autophagosome marker. Mol Biol Cell.

[17] Shvets E, Fass E, Elazar Z (2008). Utilizing flow cytometry to monitor autophagy in living mammalian cells. Autophagy.

[18] Dupont N, Leroy C, Hamai A, Codogno P (2017). Long-Lived Protein Degradation During Autophagy. Methods Enzymol.

[19] Yu ZQ, Ni T, Hong B, Wang HY, Jiang FJ, Zou S (2012). Dual roles of Atg8-PE deconjugation by Atg4 in autophagy. Autophagy.

[20] Tsuboyama K, Koyama-Honda I, Sakamaki Y, Koike M, Morishita H, Mizushima N (2016). The ATG conjugation systems are important for degradation of the inner autophagosomal membrane. Science.

[21] Itakura E, Kishi-Itakura C, Mizushima N (2012). The hairpin-type tail-anchored SNARE syntaxin 17 targets to autophagosomes for fusion with endosomes/lysosomes. Cell.

[22] Kabeya Y, Mizushima N, Ueno T, Yamamoto A, Kirisako T, Noda T (2000). LC3, a mammalian homologue of yeast Apg8p, is localized in autophagosome membranes after processing. EMBO J.

[23] Kauffman KJ, Yu S, Jin J, Mugo B, Nguyen N, O’Brien A (2018). Delipidation of mammalian Atg8-family proteins by each of the four ATG4 proteases. Autophagy.

[24] Agrotis A, Pengo N, Burden JJ, Ketteler R. Redundancy of human ATG4 protease isoforms in autophagy and LC3/GABARAP processing revealed in cells. Autophagy. 2019:15;976–97. 10.1080/15548627.2019.1569925 PMC6526816 30661429

[25] Katayama H, Kogure T, Mizushima N, Yoshimori T, Miyawaki A (2011). A sensitive and quantitative technique for detecting autophagic events based on lysosomal delivery. Chem Biol.

[26] McMurray MA, Thorner J (2008). Septin stability and recycling during dynamic structural transitions in cell division and development. Curr Biol.

[27] Takahashi Y, He H, Tang Z, Hattori T, Liu Y, Young MM (2018). An autophagy assay reveals the ESCRT-III component CHMP2A as a regulator of phagophore closure. Nat Commun.

[28] Wang JY, Yu IS, Huang CC, Chen CY, Wang WP, Lin SW (2015). Sun1 deficiency leads to cerebellar ataxia in mice. Dis Model Mech.

[29] Yang Z, Wang KK (2015). Glial fibrillary acidic protein: from intermediate filament assembly and gliosis to neurobiomarker. Trends Neurosci.

[30] Custer SK, Garden GA, Gill N, Rueb U, Libby RT, Schultz C (2006). Bergmann glia expression of polyglutamine-expanded ataxin-7 produces neurodegeneration by impairing glutamate transport. Nat Neurosci.

[31] Leranth C, Hamori J (1970). “Dark” Purkinje cells of the cerebellar cortex. Acta Biol Acad Sci Hung.

[32] Strahlendorf JC, Brandon T, Miles R, Strahlendorf HK (1998). AMPA receptor-mediated alterations of intracellular calcium homeostasis in rat cerebellar Purkinje cells in vitro: correlates to dark cell degeneration. Neurochem Res.

[33] Wang H, Bedford FK, Brandon NJ, Moss SJ, Olsen RW (1999). GABA(A)-receptor-associated protein links GABA(A) receptors and the cytoskeleton. Nature.

[34] Kneussel M, Haverkamp S, Fuhrmann JC, Wang H, Wassle H, Olsen RW (2000). The gamma-aminobutyric acid type A receptor (GABAAR)-associated protein GABARAP interacts with gephyrin but is not involved in receptor anchoring at the synapse. Proc Natl Acad Sci USA.

[35] Leil TA, Chen ZW, Chang CS, Olsen RW (2004). GABAA receptor-associated protein traffics GABAA receptors to the plasma membrane in neurons. J Neurosci.

[36] Billard JM, Vigot R, Batini CGABA (1993). THIP and baclofen inhibition of Purkinje cells and cerebellar nuclei neurons. Neurosci Res.

[37] Farrant M, Nusser Z (2005). Variations on an inhibitory theme: phasic and tonic activation of GABA(A) receptors. Nat Rev Neurosci.

[38] Egawa K, Kitagawa K, Inoue K, Takayama M, Takayama C, Saitoh S (2012). Decreased tonic inhibition in cerebellar granule cells causes motor dysfunction in a mouse model of Angelman syndrome. Sci Transl Med.

[39] Satoo K, Noda NN, Kumeta H, Fujioka Y, Mizushima N, Ohsumi Y (2009). The structure of Atg4B-LC3 complex reveals the mechanism of LC3 processing and delipidation during autophagy. EMBO J.

[40] Kyostila K, Syrja P, Jagannathan V, Chandrasekar G, Jokinen TS, Seppala EH (2015). A missense change in the ATG4D gene links aberrant autophagy to a neurodegenerative vacuolar storage disease. PLoS Genet.

[41] Fritschy JM, Panzanelli P, Kralic JE, Vogt KE, Sassoe-Pognetto M (2006). Differential dependence of axo-dendritic and axo-somatic GABAergic synapses on GABAA receptors containing the alpha1 subunit in Purkinje cells. J Neurosci.

[42] Slemmer JE, De Zeeuw CI, Weber JT (2005). Don’t get too excited: mechanisms of glutamate-mediated Purkinje cell death. Prog Brain Res.

