# Correction to: ATG4D is the main ATG8 delipidating enzyme in mammalian cells and protects against cerebellar neurodegeneration

**DOI:** 10.1038/s41418-021-00783-2

**Published:** 2021-04-23

**Authors:** Isaac Tamargo-Gómez, Gemma G. Martínez-García, María F. Suárez, Verónica Rey, Antonio Fueyo, Helena Codina-Martínez, Gabriel Bretones, Xurde M. Caravia, Etienne Morel, Nicolas Dupont, Roberto Cabo, Cristina Tomás-Zapico, Sylvie Souquere, Gerard Pierron, Patrice Codogno, Carlos López-Otín, Álvaro F. Fernández, Guillermo Mariño

**Affiliations:** 1 Departamento de Biología Funcional, Facultad de Medicina, Universidad de Oviedo, Oviedo, Spain; 2 Instituto Universitario de Oncología (IUOPA), Asturias, Spain; 3 Instituto de Investigación Sanitaria del Principado de Asturias (ISPA), Asturias, Spain; 4 Departamento de Bioquímica y Biología Molecular, Universidad de Oviedo, Oviedo, Spain; 5 Centro de Investigación Biomédica en Red de Cáncer (CIBERONC), Madrid, Spain; 6 U1151 and Institut Necker Enfants-Malades, INSERM, Sorbonne Paris Cité, Université Paris Descartes, Paris, France; 7 Departamento de Morfología y Biología Celular, Facultad de Medicina, Universidad de Oviedo, Oviedo, Spain; 8 Gustave Roussy Comprehensive Cancer Center, Villejuif, France; 9 CNRS, Villejuif, France

**Keywords:** Proteases, Macroautophagy, Disease genetics, Neural ageing, Neurological disorders

Correction to: *Cell Death & Differentiation* 10.1038/s41418-021-00776-1

The article ATG4D is the main ATG8 delipidating enzyme in mammalian cells and protects against cerebellar neurodegeneration, written by Isaac Tamargo-Gómez, Gemma G. Martínez-García, María F. Suárez, Verónica Rey, Antonio Fueyo, Helena Codina-Martínez, Gabriel Bretones, Xurde M. Caraviz, Etienne Morel, Nicolas Dupont, Roberto Cabo, Cristina Tomás-Zapico, Sylvie Souquere, Gerard Pierron, Patrice Codogno, Carlos López-Otín, Álvaro F. Fernández, Guillermo Mariño, was originally published electronically on the publisher’s internet portal on 1 April 2021 without open access. With the author(s)’ decision to opt for Open Choice the copyright of the article changed on 1 April 2021 to © The Author(s) 2021 and the article is forthwith distributed under a Creative Commons Attribution 4.0 International License, which permits use, sharing, adaptation, distribution and reproduction in any medium or format, as long as you give appropriate credit to the original author(s) and the source, provide a link to the Creative Commons licence, and indicate if changes were made.

The images or other third-party material in this article are included in the article’s Creative Commons licence, unless indicated otherwise in a credit line to the material. If material is not included in the article’s Creative Commons licence and your intended use is not permitted by statutory regulation or exceeds the permitted use, you will need to obtain permission directly from the copyright holder.

To view a copy of this licence, visit http://creativecommons.org/licenses/by/4.0/. In addition, figure 8 was printed in b/w although it was submitted in color. This has been corrected in the original article.

