# Supplementary material for: ATG4D is the main ATG8 delipidating enzyme in mammalian cells and protects against cerebellar neurodegeneration
Source: Cell Death Differ. 2021 Apr 1;28(9):2651–72. doi: 10.1038/s41418-021-00776-1 (PMC8408152; doi:10.1038/s41418-021-00776-1)
Supplement: Supplementary file 1 — Supplemental Figure legends [file 41418_2021_776_MOESM1_ESM.docx]

**Supplementary Figure Legends**

**Supplementary Figure 1. Biochemical analysis of *Atg4d*-deficient mice and quantification of immunoblots in Figure 1. (A)** Quantification of major biochemical parameters (including glucose, cholesterol, triglycerides, creatine kinase and hepatic aminotransferases) in blood samples from *WT* and *Atg4d^-/-^* mice. **(B)** Quantification of hemoglobin (HGB) and red blood cells (RBC) levels in blood samples from *WT* and *Atg4d^-/-^* mice. **(C)** Quantitative RT-PCR analysis of p62/SQSTM1 mRNA in liver tissue from *WT* and *Atg4d^-/-^* mice. (**D)** Densitometry of immunoblots in Figure 1E. (A, B and C) Bars represent mean ± SD (N > 3 mice per condition). (D) Bars represent means ± SD (N = 6 mice per genotype and condition). **P* < 0.05, 2-tailed unpaired Student’s t-test.

**Supplementary Figure 2. Quantification of immunoblots in Figure 2.** **(A)** Densitometry of immunoblots in Figure 2A. **(B)** Densitometry of immunoblots in Figure 2B. **(C)** Densitometry of immunoblots in Figure 2H. Bars represent means ± SD (N = 6 mice per genotype and condition (A). (N= 3 independent experiments (B,C) **P* < 0.05, 2-tailed unpaired Student’s t-test.

**Supplementary Figure 3. Autophagy flux analysis in *ATG4D^-/-^* HEK293T.** **(A)** Schematic representation of the CRISPR-Cas9 based introduction of a frameshift mutation into the exon 4 of ATG4D gene. **(B)** Representative images of immunofluorescence analyses of endogenous mATG8 proteins in *WT* and *ATG4D^-/-^* HEK293T and quantification of the data. **(C)** Immunoblotting analyses for autophagic flux of all human ATG8 proteins in *WT* and *ATG4D^-/-^* HEK293T cells and densitometry of the data. LOAD: β-actin. Bars represent mean ± SD (N > 80 cells per condition). Scale bars, 10 μm. **P* < 0.05, 2-tailed unpaired Student’s t test.

**Supplementary Figure 4. Degradation of isotope-labelled long-lived proteins in WT and ATG4D KO cells.** Values for cells in full medium and upon nutrient deprivation (NF) in the presence/absence of the autophagy inhibitor 3-MA in a pulse-chase analysis. Bars represent mean ± SEM (N = 6 replicates per group). **P* < 0.05, 2-tailed unpaired Student’s t test.

**Supplementary Figure 5. mATG8 proteins activation and lysosomal pH analysis**. **(A)** Schematic representation of the introduction of frameshift mutation into exon 4 of the *ATG4A* gene. **(B-F)** Control and mutant MEFs lacking ATG4D or ATG4B were transfected with mammalian expression vectors containing tagged cDNAs of mATG8s, HA-LC3A-FLAG **(B)**, 3xHA-LC3B-3xMyc **(C)**, 3xHA-GABARAP-3xMyc **(D)**, 3xMyc-GABARAPL1-3xFLAG **(E)** and 3xHA-GABARAPL2-3xMyc **(F)**. Protein extracts were subjected to immunoblotting, using ß-actin as sample processing control. Contrary to *Atg4B*-deficient cells, which show defects in mATG8s activation, ATG4D-deficient cells show comparable mATG8s activation as that observed in *WT* cells. Non-specific bands or those corresponding to putative degradation products are marked by asterisks. Note that the addition of 3xMyc epitopes (LC3B, GABARAP and GABARAPL2) or 3xFLAG (GABARAPL1) at the carboxy-terminal region causes a mobility increased in uncleaved forms of ATG8 **(G)** Representative images showing the relative fluorescence obtained from Ex329-Em440 (neutral) and Ex384-Em540 (acid) signals in *WT* and knockout MEFs cultured in the indicated conditions. Most right panels show the fluorescence intensity ratio from neutral/acidic signals. **(H)** Quantification of Lysosensor ratio from (Ex329- Em440)/(Ex384-Em540) signals, showing a lower pH in acidic organelles from *Atg4d^-/-^* cells. Each point value represents the average ratio of mKeima-LC3B positive structures of a single cell (N = 30 cells per genotype and treatment). Scale bars, 15 µm, 10 µm in insets. **P* < 0.05, 2-tailed unpaired Student’s t test. **(I).** Representative light microscopy images of H&E stained tissues from *WT* and mutant mice, showing no significantly difference between both genotypes. Scale bars, 50 µm.

**Supplementary Figure 6. Additional studies in the brain from *WT* and *Atg4d^-/-^* mice.** **(A)** Representative immunohistochemistry pictures against calbindin on cerebellum sections from 15-month-old *WT* and *Atg4d^-/-^* mice. Scale bar: 20 μm. **(B)** TUNEL assay in cerebellar sections from *WT* (up) or *Atg4d^-/-^* mice (middle). A spleen sample from an aged animal was used as a positive control. Scale bar: 200 μm. **(C)** Immunohistochemistry analyses against ubiquitin in sections form different CNS regions. Nestin-Cre/Atg7flox/flox mice are used as a positive control for ubiquitin-positive aggregates accumulation. Arrows show accumulation of poly-Ub aggregates.

**Supplementary Figure 7. Functional analysis of sex-dependent effects. (A)** Comparison of the forelimb angles against body axes in the tail suspension test. **(B)** Time course of performance on the balance beam. **(C)** Beam balance experiment scores of each group. (**D)** Quantification of stride length of *WT* and *Atg4d^-/-^* mice. **(E)** Results from grip strength analyses. **(F)** Results from Rotarod analyses. Bars represent means ± SD. (A; N=7 WT male mice, 3 WT female mice, 6 *Atg4d^-/-^* male mice and 6 *Atg4d^-/-^* female mice). (B-C; N = 13 WT male mice, 4 WT female mice, 10 *Atg4d^-/-^* male mice and 7 *Atg4d^-/-^* female mice). (D; N = 9 WT male mice, 3 WT female mice, 9 *Atg4d^-/-^* male mice and 3 *Atg4d^-/-^* female mice). (E; N = 4 WT male mice, 4 WT female mice, 4 *Atg4d^-/-^* male mice and 4 *Atg4d^-/-^* female mice). (F; N = 9 WT male mice, 3 WT female mice, 6 *Atg4d^-/-^* male mice and 9 *Atg4d^-/-^* female mice). **P* < 0.05, 2-tailed unpaired Student’s t-test.

**Supplementary Figure 8. Additional functional studies in the brain from *WT* and *Atg4d^-/-^* mice.** **(A)** Raised-bean test score. N = 17, 2-month-old and N=10, 15-month-old mice per genotype. **(B-C)** Open field activity was determined as time spent exploring the cage (B) and the center of the arena (C). **(D)** Representative trajectory of a *WT* mouse, up, and *Atg4d^-/-^* mouse, down, during open field test performance. **(E)** Long-term memory recognition index. **P* < 0.05, 2-tailed unpaired Student’s t-test (B,C and E) and two-way ANOVA followed by Dunnett´s post hoc test (A).

**Supplementary Figure 9. Additional mATG8 proteins analysis in the brain from *WT* and *Atg4d^-/-^* mice and quantification of immunoblots in Figure 5.** **(A)** Densitometry of immunoblots in Figure 5M. Bars represent means ± SD (N = 4 mice per genotype and condition). **P* < 0.05, 2-tailed unpaired Student’s t-test. **(B-C)** Representative immunoblots of endogenous ATG8-like proteins in the cortex (D) and hippocampus (E) from *WT* and *Atg4d^-/-^* mice. **(D-E)** Densitometry of immunoblots in (D) and (E) LOAD: β-actin. Bars represent means ± SD (N = 4 mice per genotype). **P* < 0.05, 2-tailed unpaired Student’s t-test.

**Supplementary Figure 10. Effects of the administration of different GABA receptors**

**modulators. (A)** Comparison of the forelimb angles against body axes in the tail suspension test before and after muscimol administration. **(B)** Rotarod experiment of *WT* and *Atg4d^-/-^* mice before and after muscimol administration. **(C)** Beam balance experiment scores of each genotype group. **(D)** Time course of performance on the balance beam before and after muscimol administration. **(E)** Comparison of the forelimb angles against body axes in the tail suspension test before and after baclofen administration. **(F)** Rotarod experiment of *WT* and *Atg4d^-/-^* mice before and after baclofen administration. **(G)** Beam balance experiment scores of each genotype group. **(H)** Time course of performance on the balance beam before and after baclofen administration. **(I)** Comparison of the forelimb angles against body axes in the tail suspension test before and after bicuculline administration. **(J)** Rotarod experiment of *WT* and *Atg4d^-/-^* mice before and after bicuculline administration. **(K)** Beam balance experiment scores of each genotype group. **(L)** Time course of performance on the balance beam before and after bicuculline administration. Bars represent means ± SD (N = 8 mice per group (A-H); N = 6 mice per genotype (I-L)). *P < 0.05, repeated measures ANOVA followed by Dunnett´s post hoc test.

**Supplementary Figure 11. Quantification of immunoblots in Figure 8.**

Densitometry of immunoblots in Figure 7C. Bars represent means ± SD (N= 3 independent experiments. **P* < 0.05, 2-tailed unpaired Student’s t-test.
