# Supplementary figures and images for: ATG4D is the main ATG8 delipidating enzyme in mammalian cells and protects against cerebellar neurodegeneration
Source: Cell Death Differ. 2021 Apr 1;28(9):2651–72. doi: 10.1038/s41418-021-00776-1 (PMC8408152; doi:10.1038/s41418-021-00776-1)

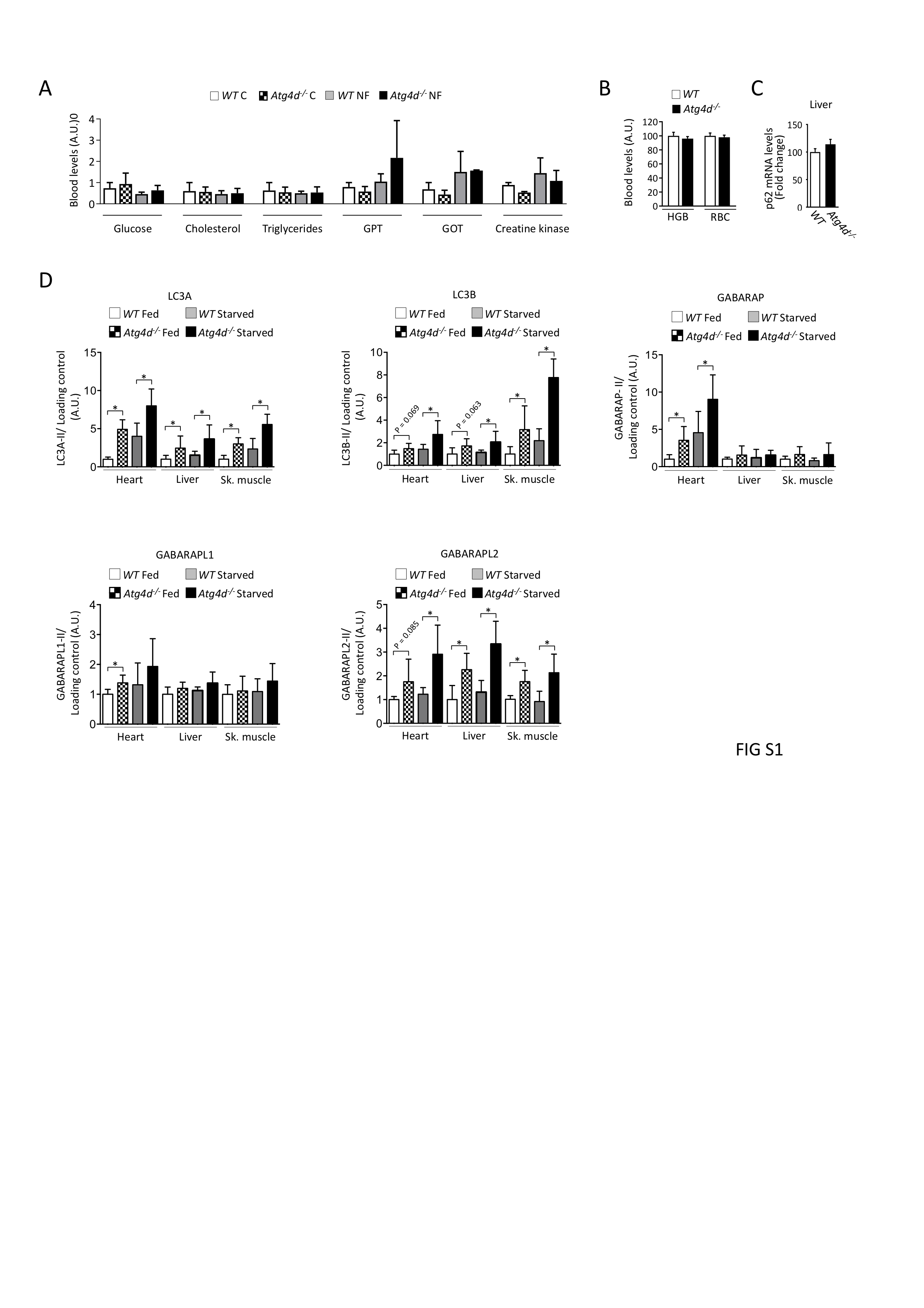

Supplement: Supplementary file 2 — Supplemental Figure 1 [file 41418_2021_776_MOESM2_ESM.png]

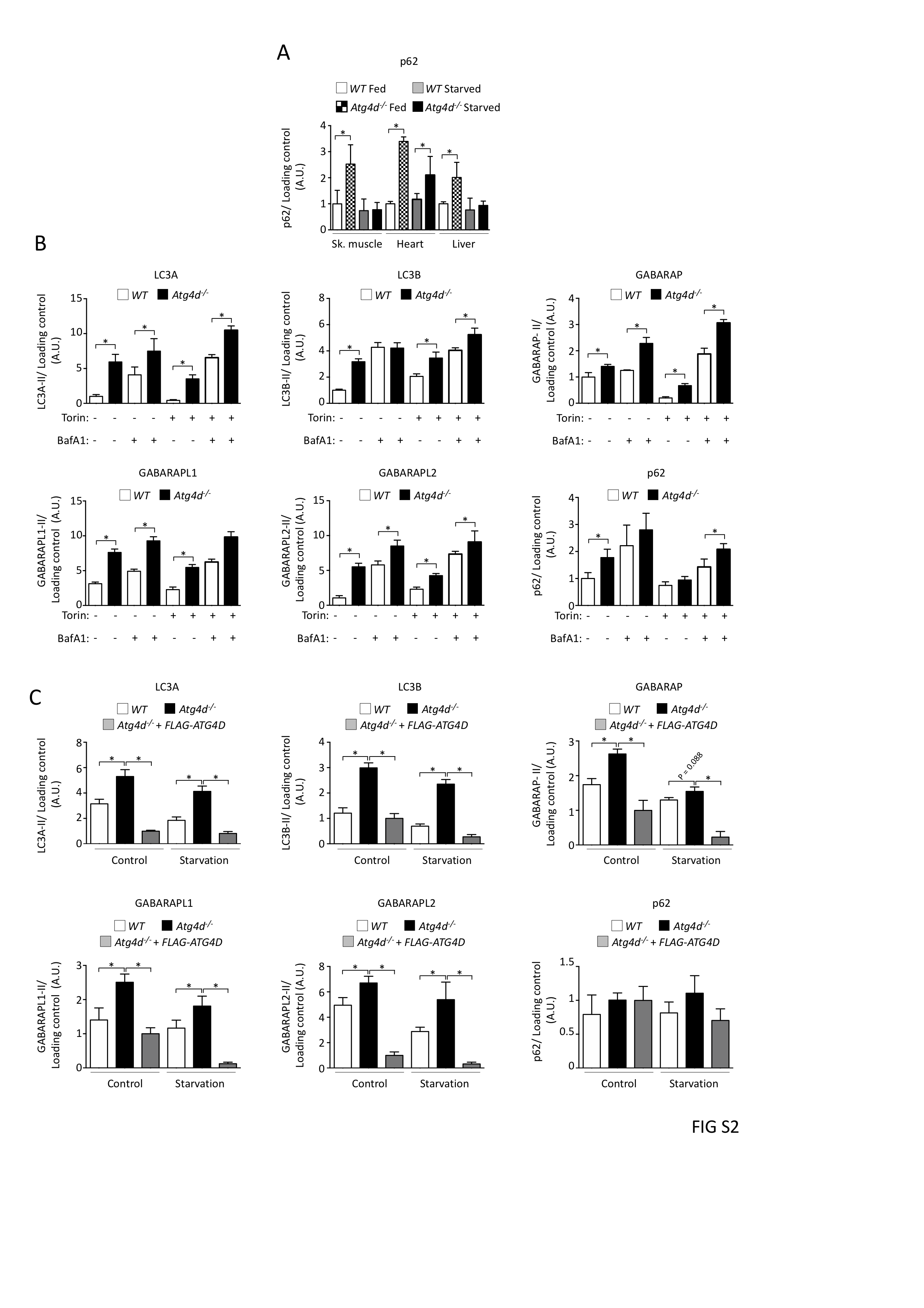

Supplement: Supplementary file 3 — Supplemental Figure 2 [file 41418_2021_776_MOESM3_ESM.png]

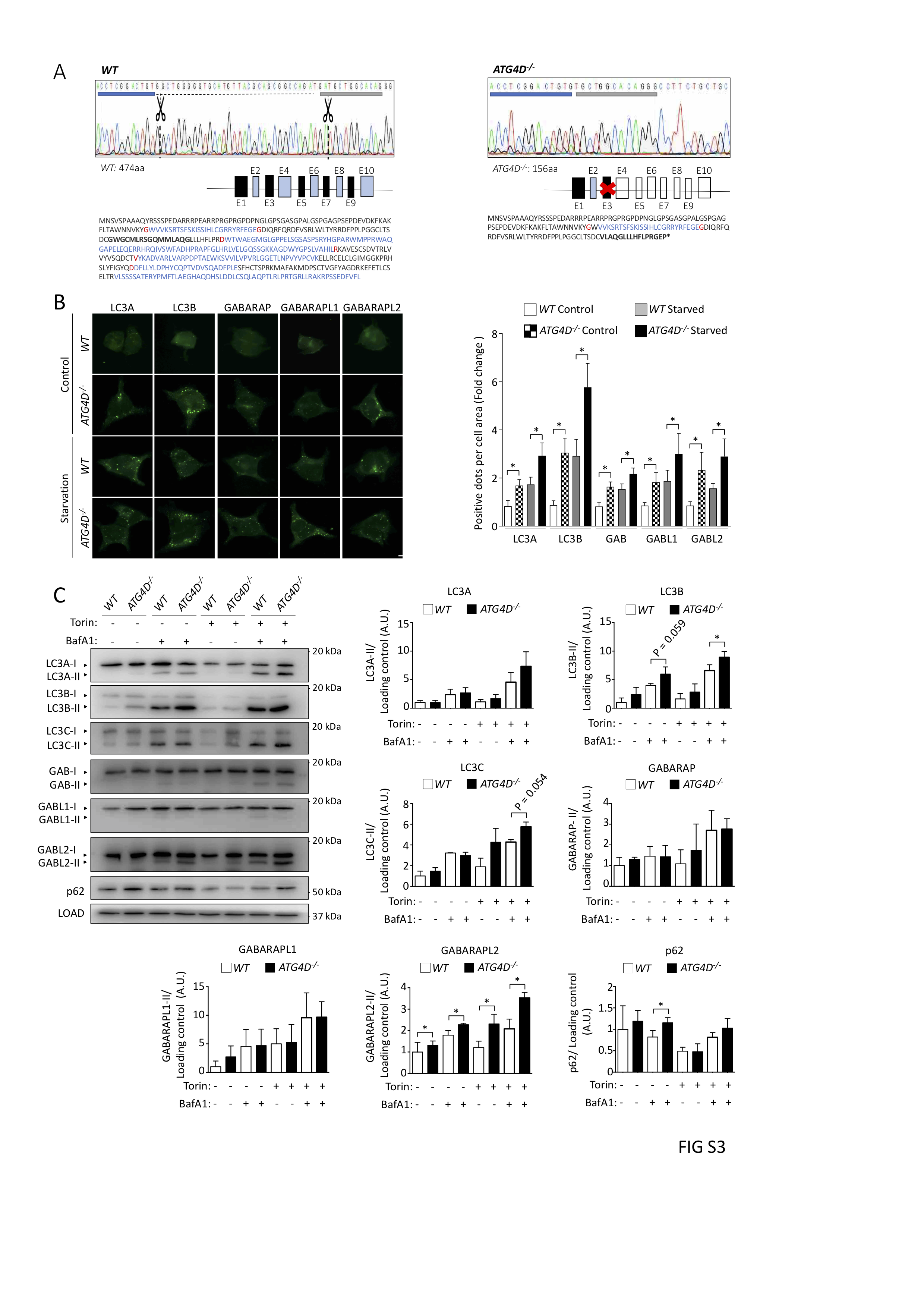

Supplement: Supplementary file 4 — Supplemental Figure 3 [file 41418_2021_776_MOESM4_ESM.png]

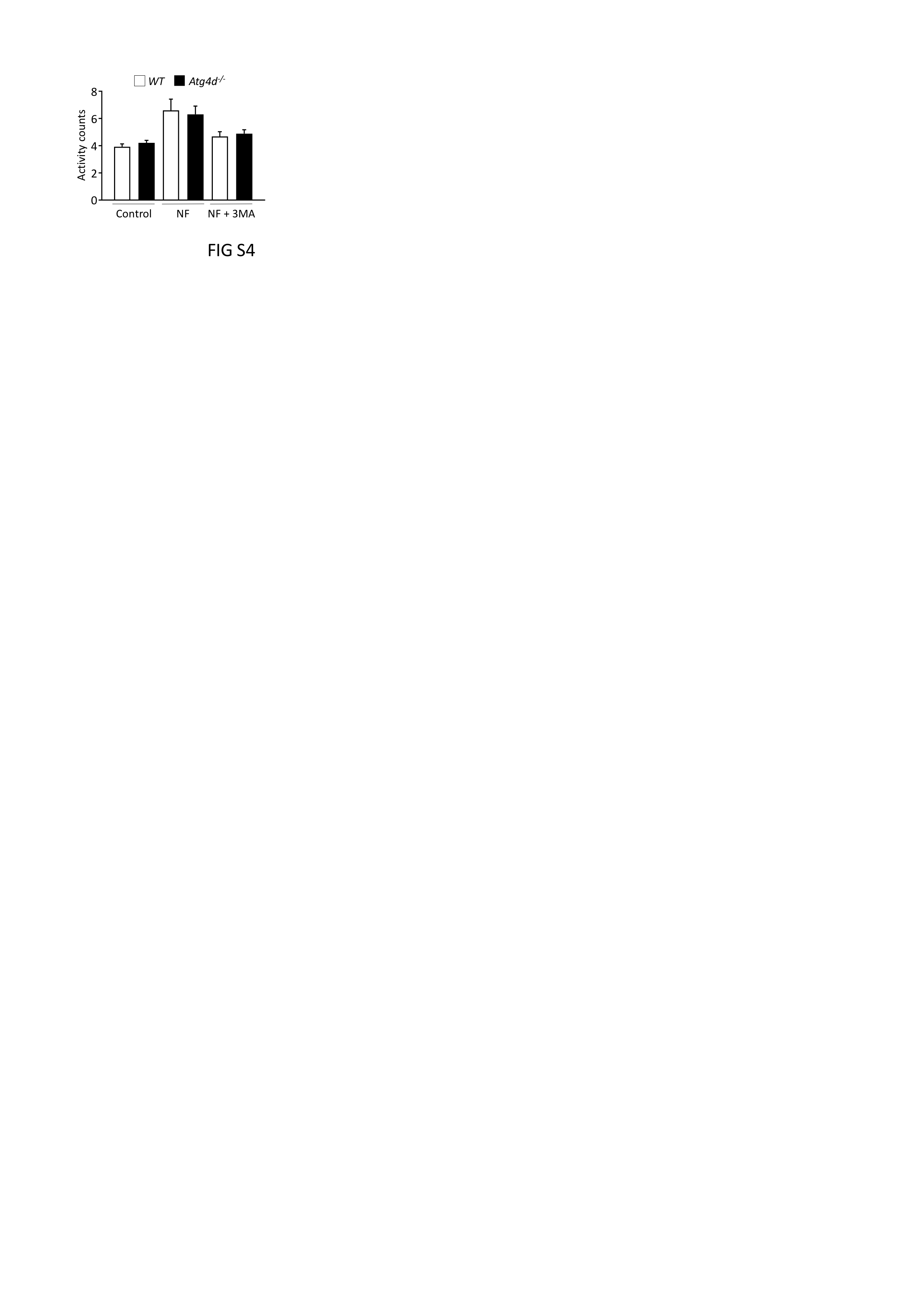

Supplement: Supplementary file 5 — Supplemental Figure 4 [file 41418_2021_776_MOESM5_ESM.png]

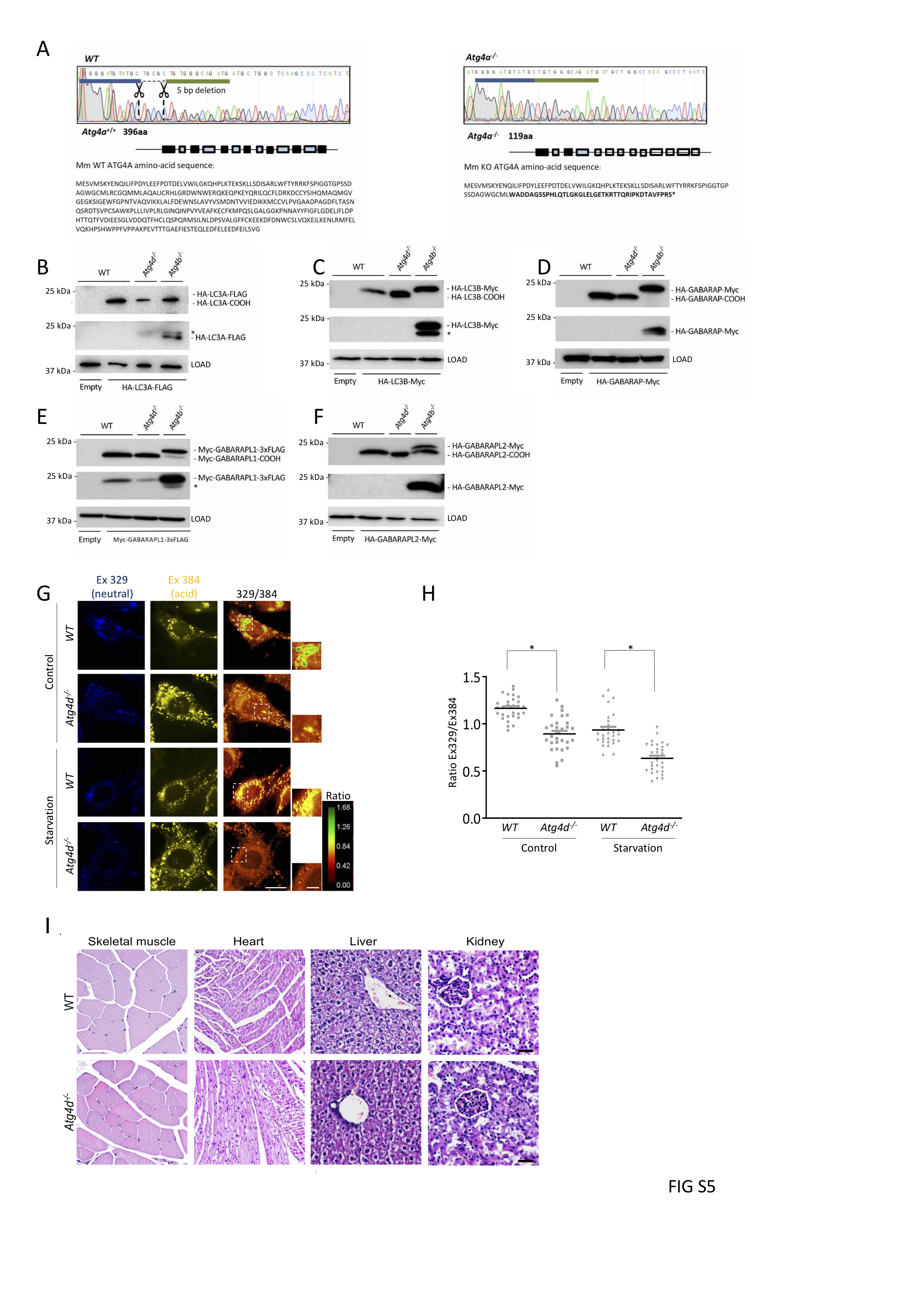

Supplement: Supplementary file 6 — Supplemental Figure 5 [file 41418_2021_776_MOESM6_ESM.png]

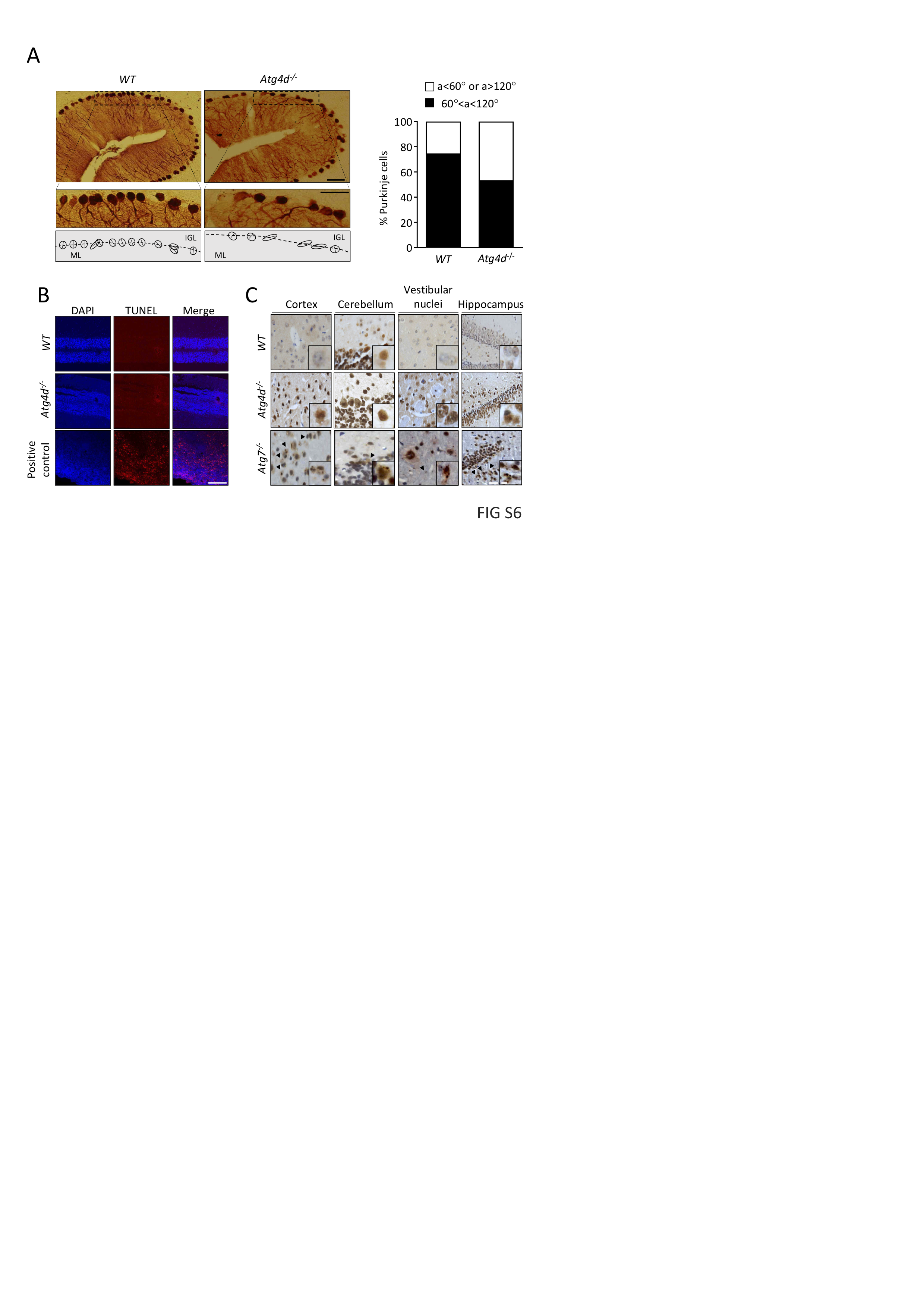

Supplement: Supplementary file 7 — Supplemental Figure 6 [file 41418_2021_776_MOESM7_ESM.png]

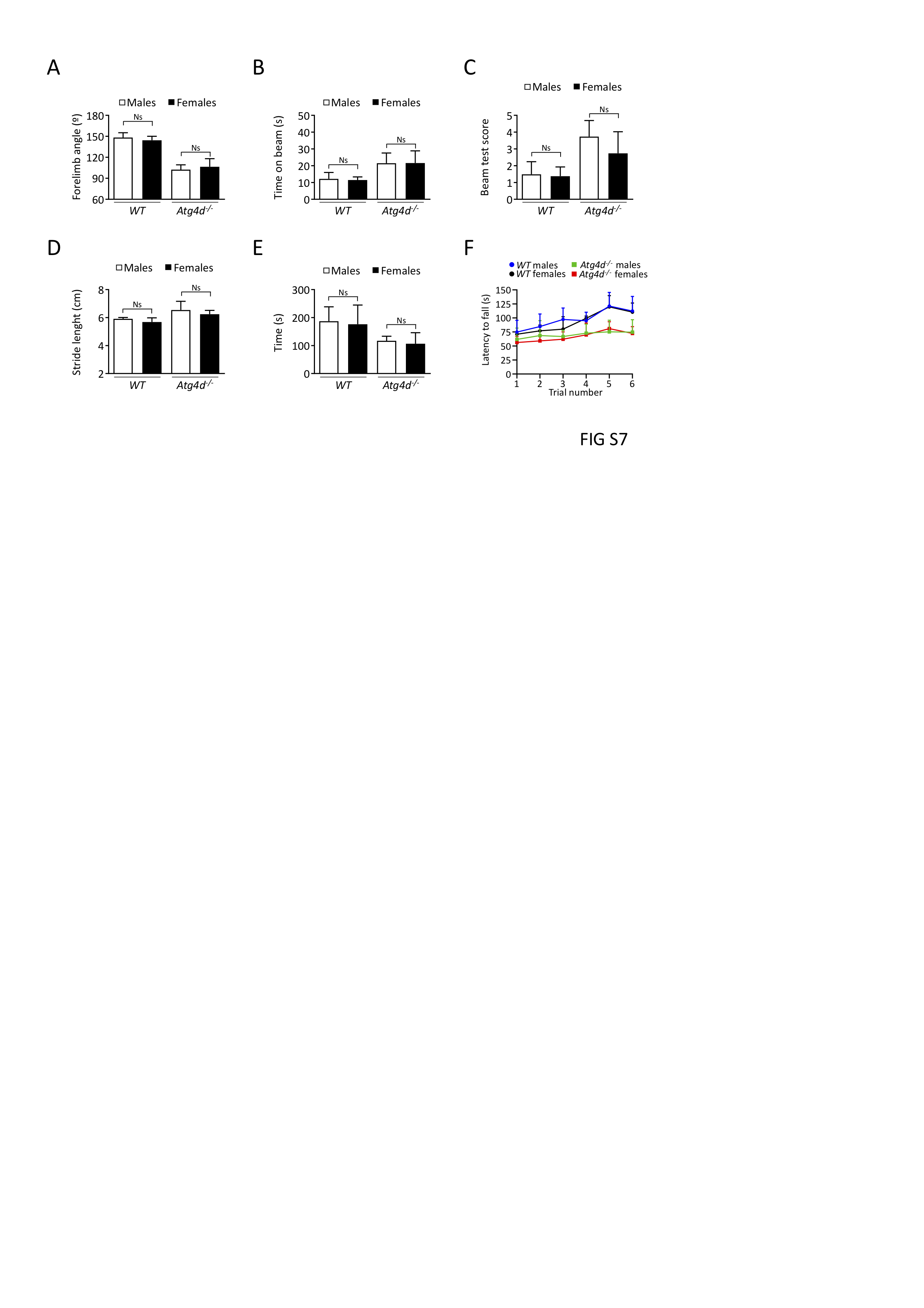

Supplement: Supplementary file 8 — Supplemental Figure 7 [file 41418_2021_776_MOESM8_ESM.png]

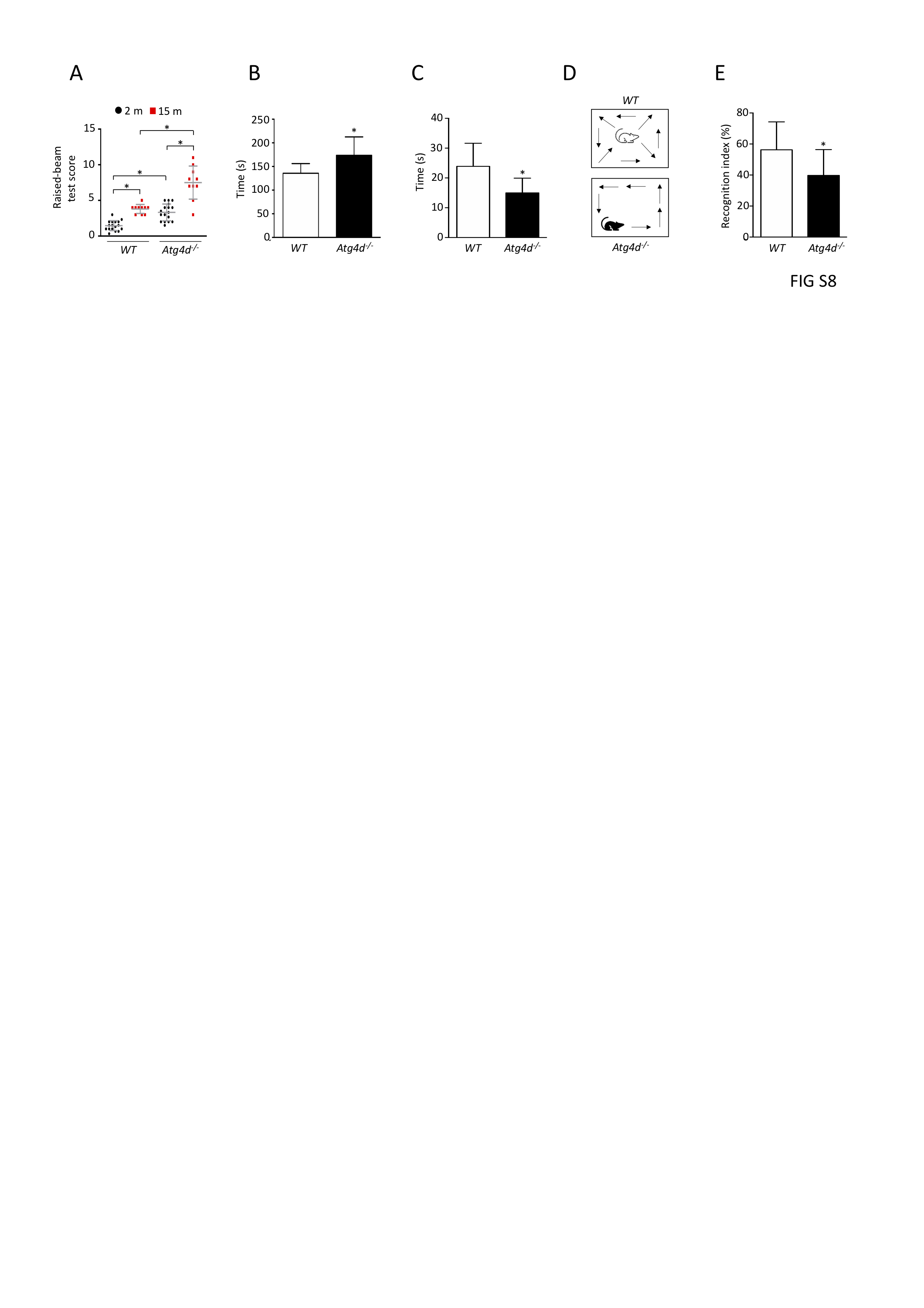

Supplement: Supplementary file 9 — Supplemental Figure 8 [file 41418_2021_776_MOESM9_ESM.png]

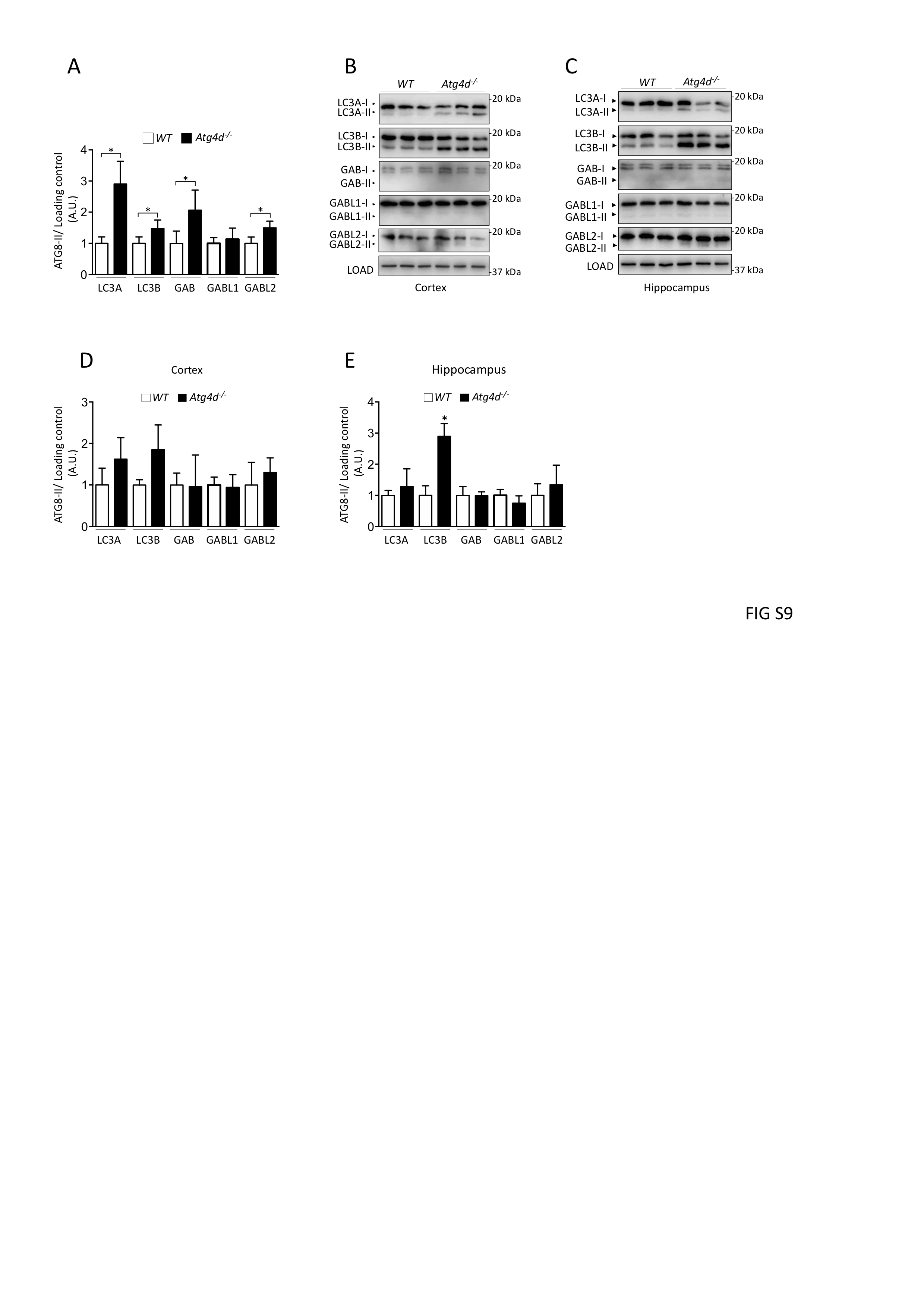

Supplement: Supplementary file 10 — Supplemental Figure 9 [file 41418_2021_776_MOESM10_ESM.png]

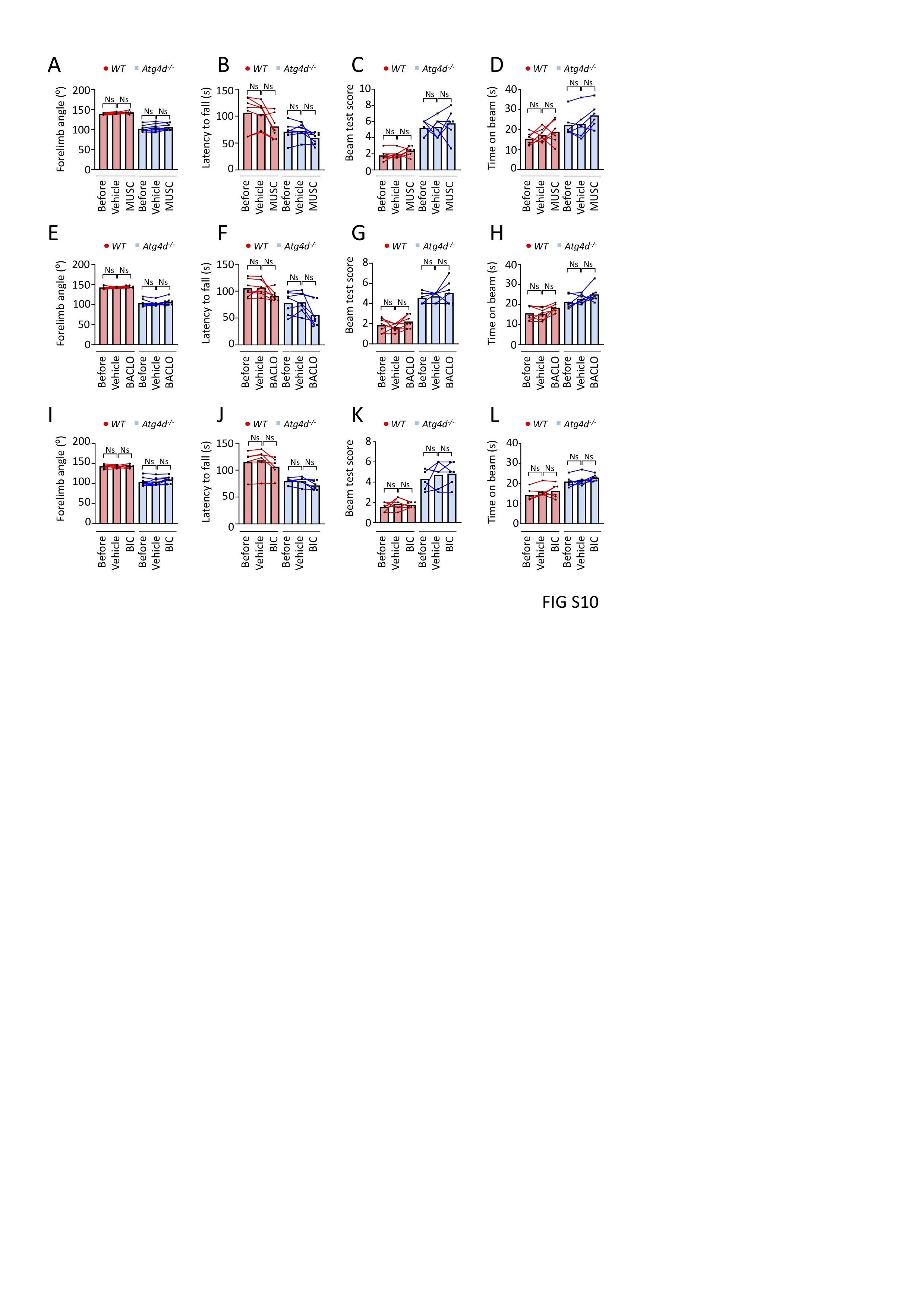

Supplement: Supplementary file 11 — Supplemental Figure 10 [file 41418_2021_776_MOESM11_ESM.png]

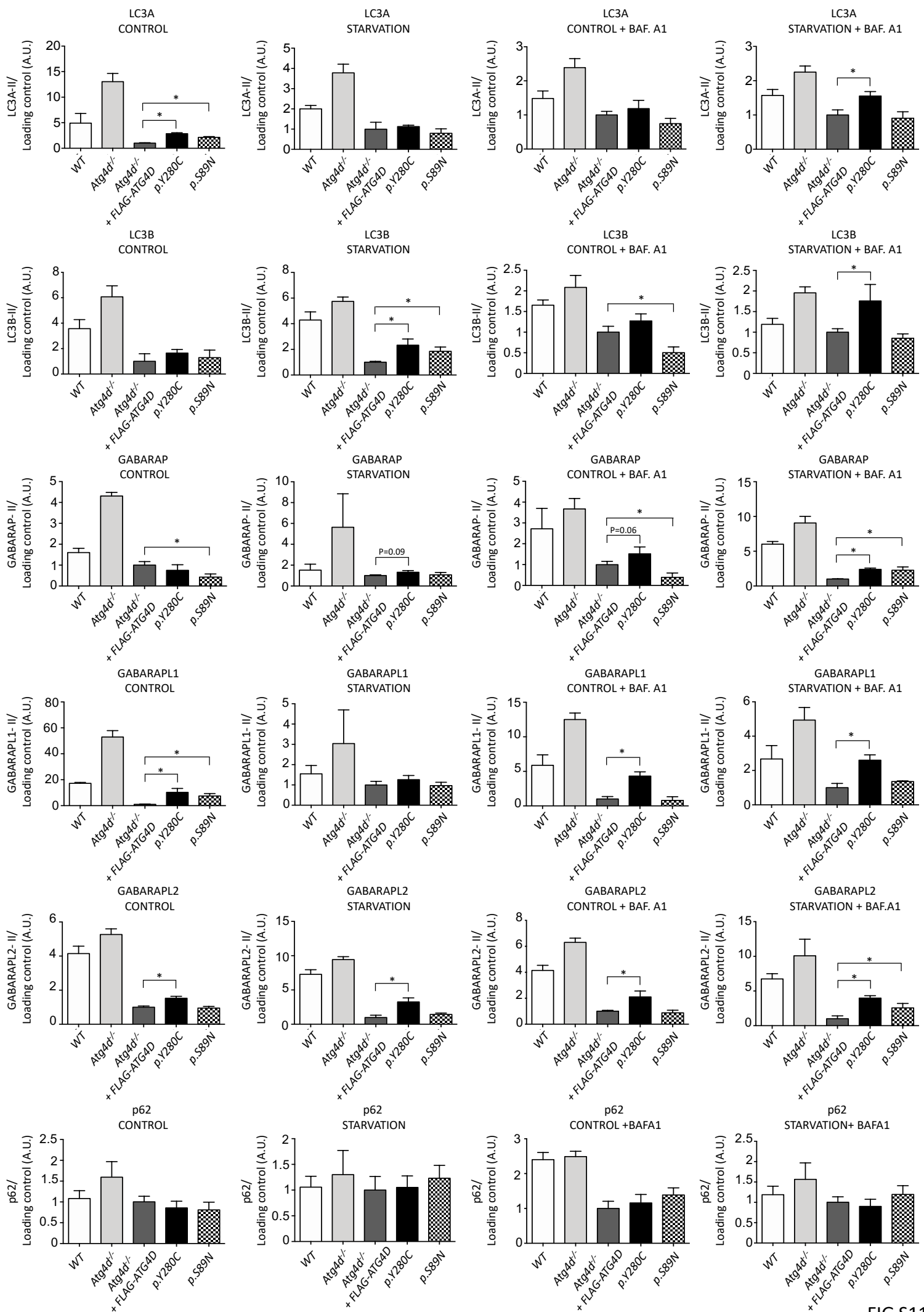

FIG S11

Supplement: Supplementary file 12 — Supplemental Figure 11 [file 41418_2021_776_MOESM12_ESM.pdf]
